# Direct relationship between protein expression and progeny yield of herpes simplex virus 1

**DOI:** 10.1128/mbio.00280-25

**Published:** 2025-05-05

**Authors:** Moeka Nobe, Yuhei Maruzuru, Kosuke Takeshima, Fumio Maeda, Hideo Kusano, Raiki Yoshimura, Takara Nishiyama, Hyeongki Park, Yoshitaka Kozaki, Shingo Iwami, Naoto Koyanagi, Akihisa Kato, Tohru Natsume, Shungo Adachi, Yasushi Kawaguchi

**Affiliations:** 1 Division of Molecular Virology, Department of Microbiology and Immunology, The Institute of Medical Science, The University of Tokyo 592607 , Minato-ku, Tokyo, Japan; 2 Department of Infectious Disease Control, International Research Center for Infectious Diseases, The Institute of Medical Science, The University of Tokyo 596903 , Minato-ku, Tokyo, Japan; 3 Research Center for Asian Infectious Diseases, The Institute of Medical Science, The University of Tokyo 592662 , Minato-ku, Tokyo, Japan; 4 Molecular Profiling Research Center for Drug Discovery (molprof), National Institute of Advanced Industrial Science and Technology (AIST) 13508, Chiyoda-ku, Tokyo, Japan; 5 Department of Proteomics, National Cancer Center Research Institute 13543 , Chuo-ku, Tokyo, Japan; 6 Interdisciplinary Biology Laboratory (iBLab), Division of Biological Science, Graduate School of Science, Nagoya University 98351 , Nagoya, Aichi Prefecture, Japan; 7 Pandemic Preparedness, Infection and Advanced Research Center, The University of Tokyo 13143, Bunkyo-ku, Tokyo, Japan; University of Colorado Anschutz Medical Campus, Aurora, Colorado, USA; Montana State University, Bozeman, Montana, USA

**Keywords:** herpes simplex virus, heterogeneity of infection, rate-limiting step for virus production

## Abstract

Although viral protein expression and progeny virus production were independently shown to be highly heterogeneous in individual cells, their direct relationship, analyzed by considering their heterogeneities, has not been investigated to date. To elucidate the direct relationship between viral protein expression and progeny virus production, we constructed a reporter herpes simplex virus 1 (HSV-1) by tagging Venus to the late protein Us11. We then separated the HSV-1-infected cell population into multiple subpopulations according to the fluorescence intensity of Venus—which reflected the expression of L proteins, largely constituting virion structural proteins—and titrated virus yields and performed electron microscopic analysis in each subpopulation. Our results revealed that infectious progeny virus production, as well as nucleocapsid maturation, was triggered only when L protein expression exceeded a specific threshold. This suggested the existence of a rate-limiting step in progeny virus production, with nucleocapsid maturation potentially being one such step.

**IMPORTANCE:**

Earlier single-cell studies of virus-infected cells have revealed high heterogeneity in the state of viral gene expression and progeny virus yield. Notably, these two aspects have been shown independently, and therefore, the direct relationship between progeny virus production and viral gene expression has been unclear. This study, for the first time, demonstrated the direct and quantitative relationship between viral protein expression and progeny virus production by taking into account their heterogeneities and revealed a threshold for the levels of herpes simplex virus 1 protein expression for progeny virus production, thereby suggesting the existence of a rate-limiting step in progeny virus production.

## INTRODUCTION

The state of viral gene expression has long been thought to be one of the critical determinants for virus progeny production (1). Viral infection is usually studied at the entire population level by averaging the outcomes of infection from each of large numbers of individual cells. In these previous studies, a relationship between viral gene expression and virus progeny production has necessarily been investigated based on classical time course experiments in which levels of viral gene expression and progeny virus titers were compared at various times after infection. Such studies demonstrated that the levels of viral gene expression, including the expressions of viral mRNA and protein, correlated well with progeny virus yields (2–6). However, accumulating evidence has indicated that viral infection at the single-cell or subpopulation level is highly heterogeneous, which has been masked in studies performed at the entire population level. Thus, it was reported that the progeny virus yield from individual cells spanned several orders of magnitude (7–14). Classical fluorescence microscopy and recent advances in single-cell RNA sequencing have enabled the investigation of the state of viral gene expression at the single-cell level, revealing high heterogeneity in individual cells and identifying new subpopulations of infected cells with similar viral gene expression profiles (15–17). However, there is a lack of information on viral protein expression and virus progeny production in the same individual cell or subpopulation. Therefore, the direct relationship between viral protein expression and progeny virus production, analyzed by considering their heterogeneities at single-cell or subpopulation levels, remains to be elucidated. These observations raised a fundamental question as to whether the well-established correlation between viral protein expression and progeny virus production detected at the entire population level by classical time-course experiments (4, 6) reflects a direct relationship between them.

Herpes simplex virus 1 (HSV-1), an extensively studied DNA virus, is a ubiquitous human pathogen, causing a variety of diseases including encephalitis, keratitis, and mucocutaneous and skin diseases including herpes labialis, genital herpes, and herpetic whitlow (18). HSV-1 encodes more than 100 different proteins (19–21) and HSV-1 genes fall into three major classes: immediate-early (IE), early (E), and late (L), whose expressions are coordinately regulated and sequentially ordered in a cascade during lytic infection (19). Although there are some exceptions, virion structural proteins are largely encoded by L genes (19). Replication of the HSV-1 genome, formation of capsids, and packaging of the replicated viral genomes into nascent capsids occurs in the nucleus (19). Thus, the immature capsid, termed the procapsid, is generated through the association between a complex comprising VP5 and scaffolding proteins UL26.5 and UL26, along with a triplex complex formed by VP23 and VP19c, in conjunction with portal protein UL6 (22–24). Following procapsid formation, the proteolytic activity of UL26 is activated, resulting in the disassociation of the scaffolding proteins from the capsid shell (22–24). Subsequently, the viral genome is encapsulated, with viral genome transport into the capsid mediated by the viral terminase, a three-component ATPase complex composed of UL15, UL28, and UL33 (22–24). The HSV-1 terminase cleaves nascent viral concatemeric DNA into unit-length genomes, docks at the capsid portal vertex composed of UL6, and packages the cleaved progeny virus genome into the capsid (22–24). Nascent nucleocapsids are exported to the cytoplasm through the perinuclear space between the inner nuclear membrane and outer nuclear membrane by a nuclear pore-independent and sequential envelopment/de-envelopment process (19). In the cytoplasm, capsids acquire a final envelope by budding into cytoplasmic vesicles and become infectious (19). Mature virions then are secreted from the infected cells by exocytosis (19). The L genes also encode proteins required for these virion maturation processes.

In this study, we constructed a genetically engineered fluorescent reporter HSV-1 to monitor the global expression of viral L proteins. We separated the entire infected cell population into multiple subpopulations based on their fluorescence intensity and determined virus titers in each subpopulation, enabling us to elucidate the direct relationship between HSV-1 L protein expression and progeny virus production. The clarified relationship indicated a threshold in the levels of HSV-1 L protein expression for progeny virus production, suggesting the existence of a rate-limiting step for progeny virus production.

## RESULTS

### Construction and characterization of a reporter HSV-1 to analyze viral L protein expression and virus progeny yields at the subpopulation level

**Fig 1:**
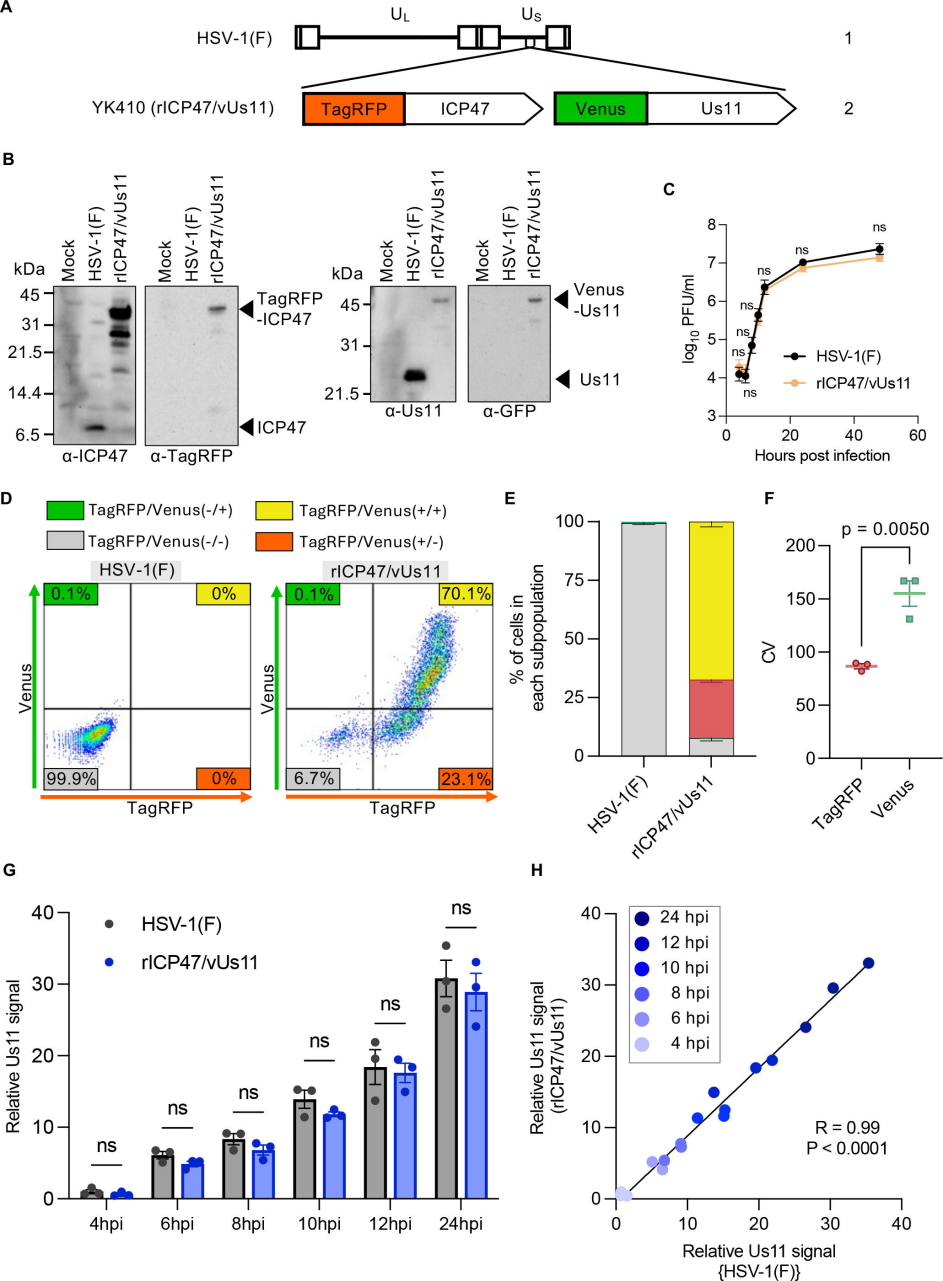
Characterization of the reporter HSV-1 rICP47/vUs11 generated in this study. (**A**) Schematic diagram of the genome structure of wild-type HSV-1(F) and rICP47/vUs11. Line 1, wild-type HSV-1(F) genome; line 2, domains of the Us11 and ICP47 coding regions. The positions of insertion of TagRFP and Venus are indicated. (**B**) HeLa cells mock-infected or infected for 24 h with wild-type HSV-1(F) or rICP47/vUs11 at an MOI of 5 were lysed and analyzed by immunoblotting with the indicated antibodies. (**C**) HeLa cells were infected at an MOI of 5 with wild-type HSV-1(F) or rICP47/vUs11. Each cell culture supernatant, along with the infected cells, was harvested at the indicated times post-infection, and progeny viruses were assayed on Vero cells. (**D**) HeLa cells infected for 24 h with wild-type HSV-1(F) or rICP47/vUs11 at an MOI of 5 were analyzed by flow cytometry. (**E**) Quantitative bar graph of the proportion of cells in the TagRFP/Venus (+/+), TagRFP/Venus (+/−), TagRFP/Venus (−/+), and TagRFP/Venus (−/−) subpopulations shown in panel **D**. (**F**) CV values for TagRFP-ICP47 and Venus-Us11 are shown in panel **D**. (**G and H**) HeLa cells were infected with wild-type HSV-1(F) or rICP47/vUs11 at an MOI of 5. At 4, 6, 8, 10, 12, and 24 h post-infection, the infected cells were detached from culture plates, fixed, permeabilized, and then incubated with rabbit polyclonal anti-Us11 antibody. After washing, the cells were incubated with anti-rabbit IgG conjugated to Alexa Fluor 647, washed again, and subsequently analyzed by flow cytometry. (**G**) Bar graph showing the relative Us11 signal (Alexa Fluor 647) of HSV-1(F)-infected and rICP47/vUs11-infected cells at 4, 6, 8, 10, 12, and 24 h post-infection. (**H**) Correlation of the relative Us11 signal (Alexa Fluor 647) between HSV-1(F)-infected and rICP47/vUs11-infected cells. Each point represents an individual result from three independent experiments. The data are representative of three independent experiments (**B and D**). Each value is the mean ± SE of the results of three (C, E, F, and G) independent experiments. Statistical analysis was performed using an unpaired Student’s *t*-test. ns, not significant (**C, F, and G**).

We constructed a recombinant virus rICP47/vUs11 expressing IE protein ICP47 and L protein Us11 fused to monomeric fluorescent proteins, TagRFP and VenusA206K (TagRFP-ICP47 and Venus-Us11), respectively (Fig. 1A and B). The growth of rICP47/vUs11 was similar to wild-type HSV-1(F) in HeLa cells at a multiplicity of infection (MOI) of 5 and reached a plateau 24 h after infection (Fig. 1C). Flow cytometric analyses showed 92% of HeLa cells inoculated with rICP47/vUs11 at an MOI of 5 were TagRFP positive 24 h after inoculation, indicating that in these experimental settings, most HeLa cells were infected with rICP47/vUs11, and HSV-1 gene expression was initiated in these infected cells (Fig. 1D and E). Frequencies of cells positive for Venus and TagRFP, or those positive for TagRFP and negative for Venus, were 67% and 25%, respectively (Fig. 1E). Cells positive for Venus and negative for TagRFP were barely detectable (Fig. 1D and E). Notably, the coefficient of variation (CV) for Venus-Us11 fluorescence intensities in each infected cell was significantly higher than for TagRFP-ICP47 fluorescence (Fig. 1F), indicating that L proteins in particular exhibited highly heterogeneous protein expression in individual cells. To verify whether the expression kinetics of Venus-Us11 in rICP47/vUs11-infected cells are similar to the expression kinetics of Us11 in wild-type virus-infected cells, we collected HSV-1(F)- and rICP47/vUs11-infected cells at various time points, then fixed, permeabilized, and stained them with an anti-Us11 antibody for flow cytometry. As shown in Fig. 1G and H, the temporal expression profiles of Us11 (or Venus-Us11) were comparable in both infections, indicating that the L protein expression kinetics of rICP47/vUs11 was equivalent to that of wild-type HSV-1(F). HSV-1 L proteins include virion structural proteins and those required for virion maturation processes, suggesting that the expression of these viral L proteins might be directly linked to virus progeny production. In this study, we separated the entire infected cell population into six subpopulations based on Venus-Us11 fluorescence intensity and determined virus titers in each subpopulation to elucidate the direct relationship between HSV-1 L proteins and progeny virus production.

**Fig 2:**
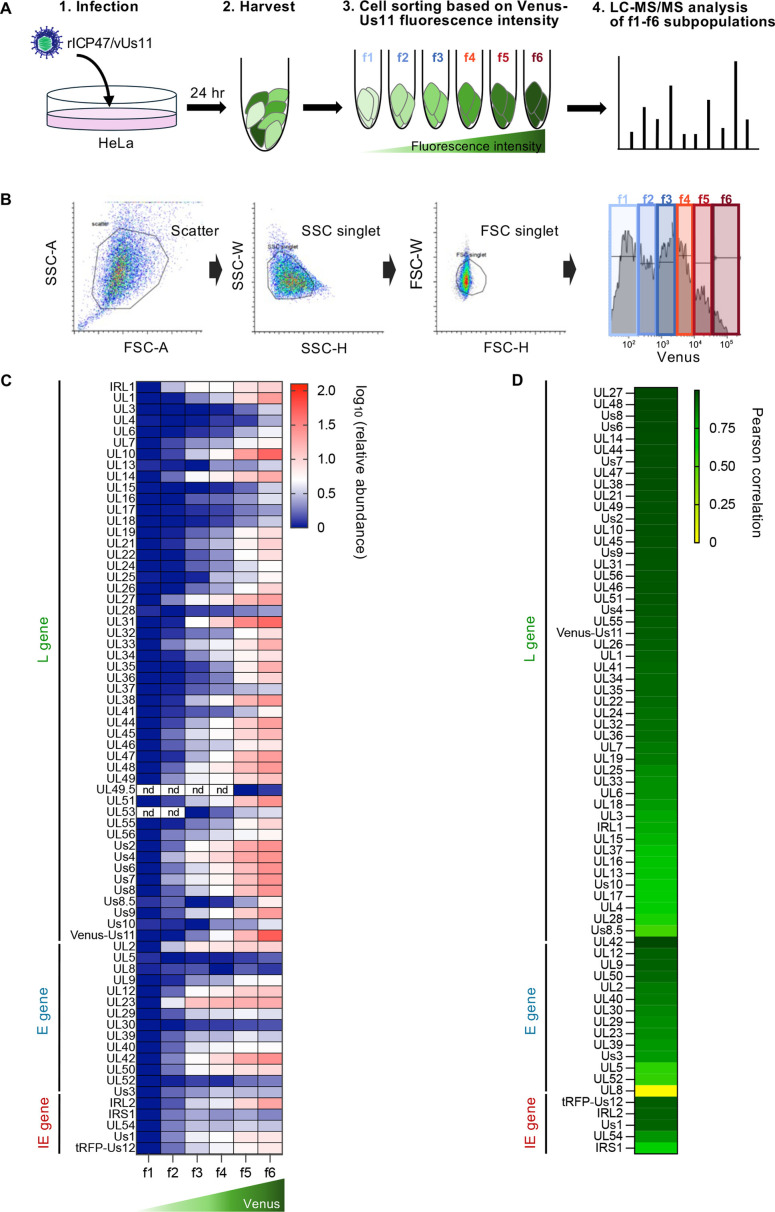
Abundance of HSV-1 L proteins in each subpopulation of rICP47/vUs11-infected cells. (**A**) Schematic of the experimental workflow. HeLa cells were infected with rICP47/vUs11 at an MOI of 5, harvested 24 h post-infection, and sorted into six subpopulations (f1–f6) based on Venus-Us11 fluorescence intensity. Each fraction was subsequently subjected to LC-MS/MS analysis. (**B**) Gating strategy corresponding to the workflow in panel **A**. The same gates were consistently applied to all other experiments involving cell sorting in this study. (**C**) The heatmap of log_10_ (relative abundance) of 69 HSV-1 proteins in each subpopulation is shown. Data are the mean values from three biologically independent experiments. nd, not detected. (**D**) Heatmap of the Pearson correlation coefficient between log_10_ (mean fluorescent intensity [MFI] of Venus) and log_10_ (relative abundance) of 67 HSV-1 proteins across the f1–f6 subpopulations. The data were based on those presented in S-Fig. 2A. *P*-values for the correlation coefficient of all HSV-1 L proteins except UL8 shown in panel **D** were <0.05.

To examine whether levels of Venus-Us11 fluorescence in rICP47/vUs11-infected cells were related to those of the global expression of HSV-1 L proteins, we used a shotgun proteomics approach to comprehensively quantitate the relative abundance of global HSV-1 and cellular proteins in infected cells. Thus, HeLa cells infected with rICP47/vUs11 at an MOI of 5 for 24 h were analyzed by flow cytometry and sorted into six subpopulations (f1–f6) based on the levels of Venus-Us11 fluorescence intensities—Venus-Us11 fluorescence intensities increased in subpopulations from f1 to f6 (Fig. 2A and B). The subpopulations were then subjected to liquid chromatography-tandem mass spectrometry (LC-MS/MS) to quantify global HSV-1 and cellular proteins in infected cells (Fig. 2C; Fig. S1 and S2A). The reproducibility of the three biological replicates was confirmed because the proteins detected by mass spectrometry were similar across all fractions (Fig. S1A), and the correlation coefficients of the detected protein abundances were high, ranging from 0.82 to 0.99 for detected host and viral proteins (Fig. S1B). Among the 69 HSV-1 proteins detected, 66 proteins were consistently detected across all the 18 data points (six subpopulations × three replicates), and Us8.5 was detected in 17 out of the 18 data points. In contrast, UL53 and UL49.5 were each detected in only 4 of the 18 data points, and thus, the data points for UL53 and UL49.5 were excluded from further analysis.

To quantitatively analyze the relationship between Venus-Us11 fluorescence intensity and the global expression of HSV-1 proteins, we calculated the Pearson correlation coefficients between the levels of Venus-Us11 fluorescence intensity and the relative abundance of 67 HSV-1 proteins across the six subpopulations (f1–f6; Fig. 2D). Among the HSV-1 L proteins analyzed (48 L proteins), there was a strong correlation between the levels of Venus-Us11 fluorescence intensity across the subpopulations f1–f6 and the relative abundance of most HSV-1 L proteins including Venus-Us11 (31 L proteins, *r* > 0.90, *P* < 0.0001; 12 L proteins, *r* > 0.70, *P* < 0.001; 5 L proteins, *r* > 0.53, *P* < 0.05). We should note that the levels of Venus-Us11 fluorescence intensity across the f1–f6 subpopulations also correlated with the relative abundance of all detected IE and E proteins (3 IE proteins, *r* > 0.90, *P* < 0.0001; 1 IE protein, *r* > 0.70, *P* < 0.0001; 1 IE protein, *r* > 0.60, *P* < 0.01; 4 E proteins, *r* > 0.90, *P* < 0.0001; 7 E proteins, *r* > 0.70, *P* < 0.0001; 2 E proteins, *r* > 0.57, *P* < 0.05) but not UL8 (*r* = 0.0005, *P* = 1.0; Fig. 2D). A comparison of the correlation coefficients of IE, E, and L indicated no statistically significant differences in *r* values for IE and E proteins among subpopulations f1–f6 or f1–f4 (Fig. S2B). In contrast, the *r* values for IE and E proteins in subpopulations f4–f6 were significantly lower than those for L proteins (Fig. S2B). These results suggested that Venus-Us11 fluorescence intensities more accurately reflected expression levels of global HSV-1 L proteins than those of IE and E proteins in rICP47/vUs11-infected cells.

### Specific Venus-Us11 protein expression levels are linked to progeny virus production

**Fig 3:**
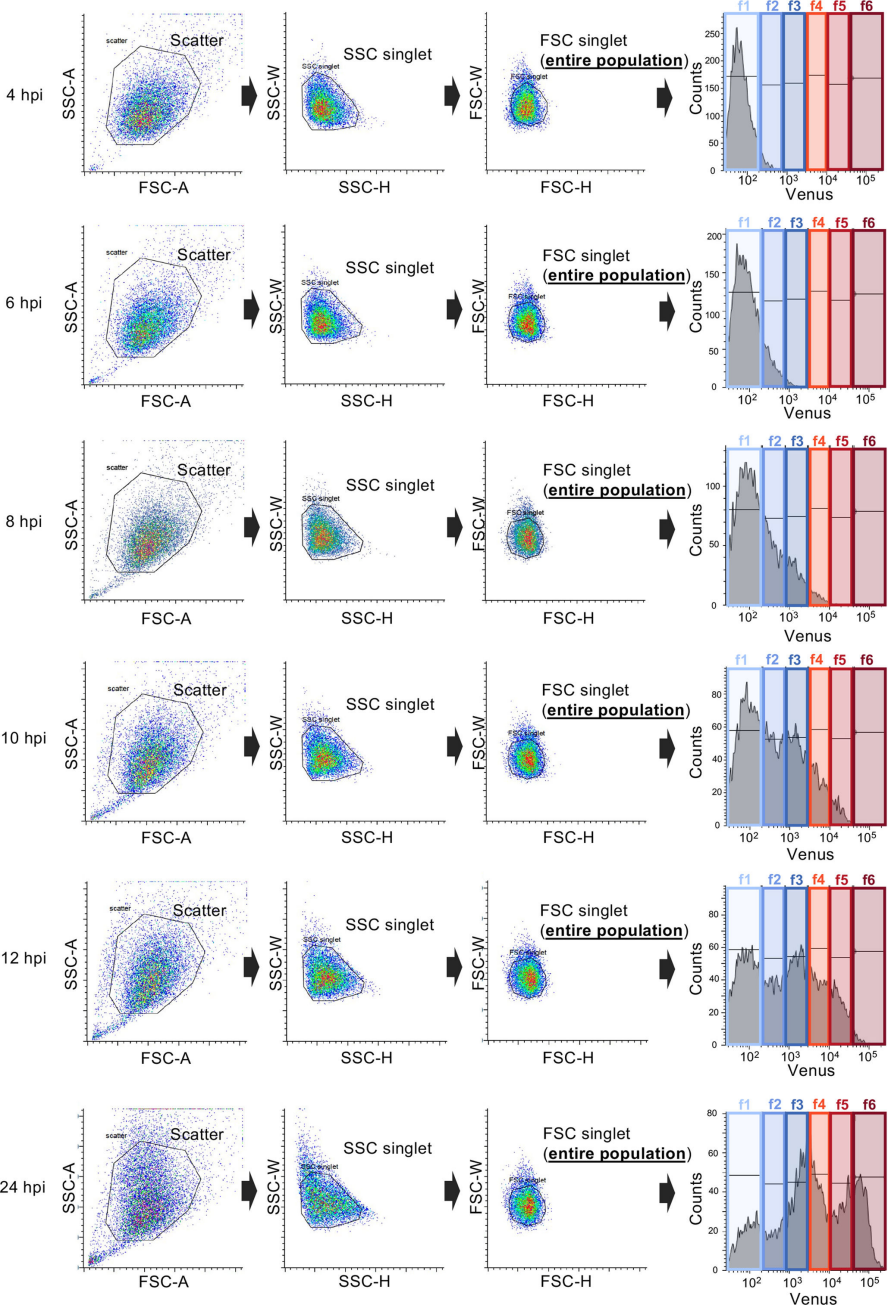
Gating strategy for the experiments shown in Fig. 4. HeLa cells were infected with rICP47/vUs11 at an MOI of 5, analyzed by flow cytometry, and sorted into an entire cell population (FSC singlet) or f1–f6 subpopulations by cell sorting at the indicated times after infection. Sorted cells were analyzed as shown in Fig. 4.

To analyze a relationship between expression levels of HSV-1 L proteins and progeny virus yields directly and quantitatively, HeLa cells were infected with rICP47/vUs11 at an MOI of 5, harvested at 4, 6, 8, 10, 12, and 24 h after infection, analyzed by flow cytometry, and sorted into entire population (FSC singlet) or six subpopulations (f1–f6) according to the levels of Venus-Us11 fluorescence intensity (Fig. 3). Virus titers in the entire population and each of the subpopulations were determined, and virus titers per 10^4^ cells were estimated. The Venus-Us11 fluorescence intensities of each subpopulation at 24 h post-infection were consistent with those obtained in the mass spectrometry analysis (Fig. S3A), verifying that our experimental system consistently collected cell populations with comparable levels of viral L protein expression across independent experiments.

**Fig 4:**
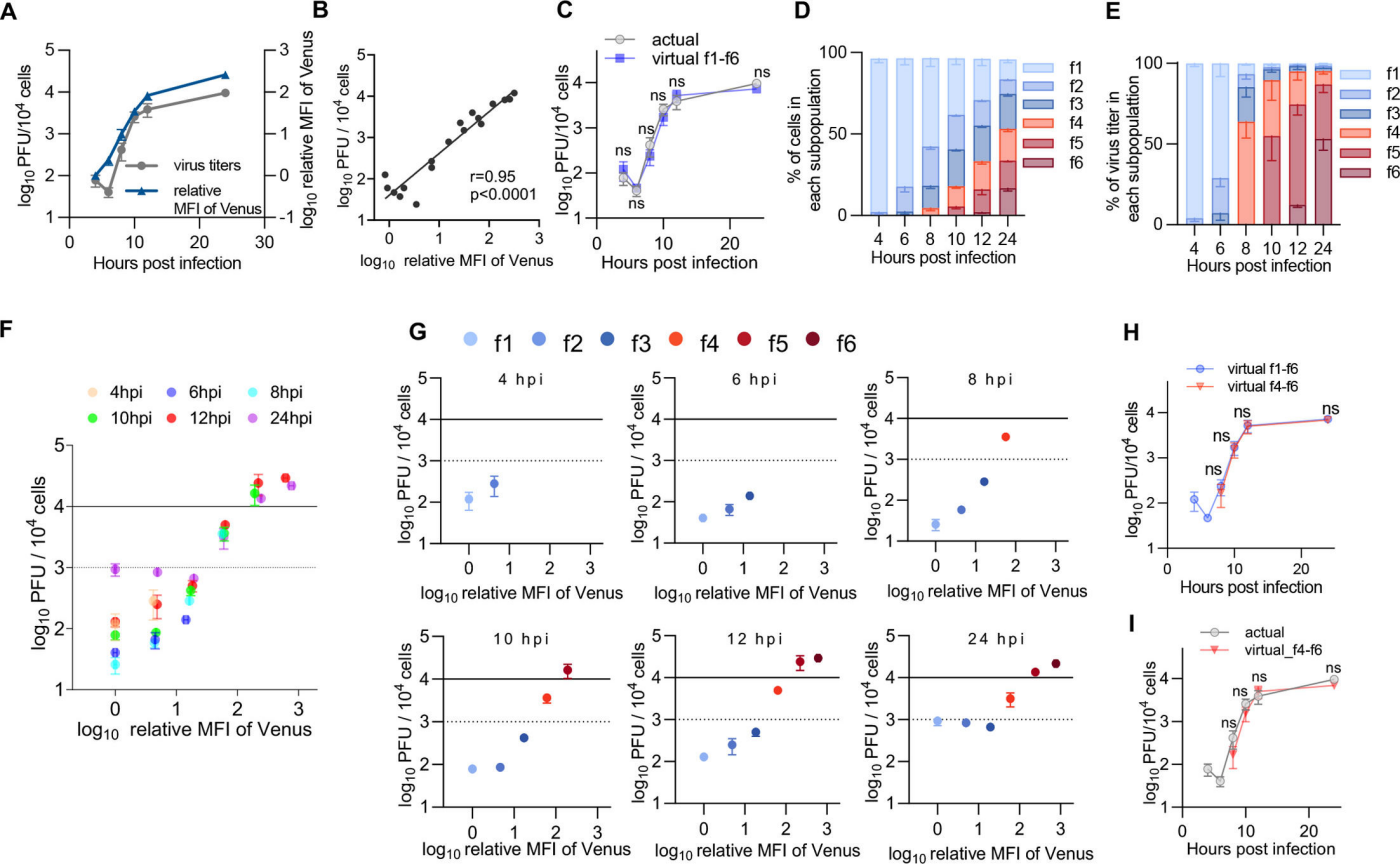
Quantitative analysis of a relationship between the expression levels of HSV-1 L proteins and progeny virus yields. (A–I) HeLa cells were infected with rICP47/vUs11 at an MOI of 5 and sorted as shown in Fig. 3. Sorted cells were sonicated, and virus titers were determined by plaque assay using Vero cells. (**A**) Kinetics of mean fluorescent intensity (MFI) of Venus and progeny virus titers of the entire population. (**B**) Scatter plot of log_10_(relative MFI of Venus) vs log_10_(plaque-forming unit [PFU]/10^4^ cells) from panel A. (**C**) Kinetics of progeny virus titers of the entire population from panel A (actual) and the sum of the virus titers produced by f1–f6 subpopulation (virtual_f1-f6). The calculation method for virtual titers is described in Materials and Methods. (**D**) Proportion of cells in the indicated subpopulation accounts for the entire cell population at the indicated times after infection. (**E**) Proportion of virus titers produced by the f1–f6 subpopulations accounts for virus titers produced by the entire population at the indicated times after infection. (**F**) Scatter plots of log_10_(relative MFI of Venus) vs log_10_(PFU/10^4^ cells). (**G**) Scatter plots of log_10_(relative MFI of Venus) vs log_10_(PFU/10^4^ cells) of the indicated subpopulations separated by time after infection from panel F. (**H**) Kinetics of the sum of the virus titers produced by f1–f6 (virtual_f1-f6) or f4–f6 (virtual_f4-f6) subpopulations. (**I**) Kinetics of progeny virus titers of the entire population from panel A (actual) and the sum of the virus titers produced by f4–f6 subpopulations (virtual_f4-f6). Each value is the mean ± SE (A and C–I) or individual value (**B**) of the results of three independent experiments. Statistical analysis was performed by unpaired Student’s *t*-test. ns, not significant (C, H, and I). Solid and dashed lines indicate 1 PFU/cell and 0.1 PFU/cell, respectively (**F and G**).

In agreement with previous reports (4, 6), the kinetics of Venus-Us11 fluorescence intensity were similar to those of progeny virus titers (Fig. 4A), and Venus-Us11 fluorescence intensities had a high correlation with progeny virus titers at the entire population level (*r* = 0.95; Fig. 4B). We also calculated virtual titers (VTs) per 10^4^ cells at each time point by summing virus titers in f1–f6 subpopulations, obtained by multiplying the estimated virus titer of each subpopulation of 10^4^ cells by the ratio of the number of cells in the subpopulation to that in the entire population. A viral growth curve based on the virtual titers was almost identical to that based on actual titers in the entire population (Fig. 4C).

**Fig 5:**
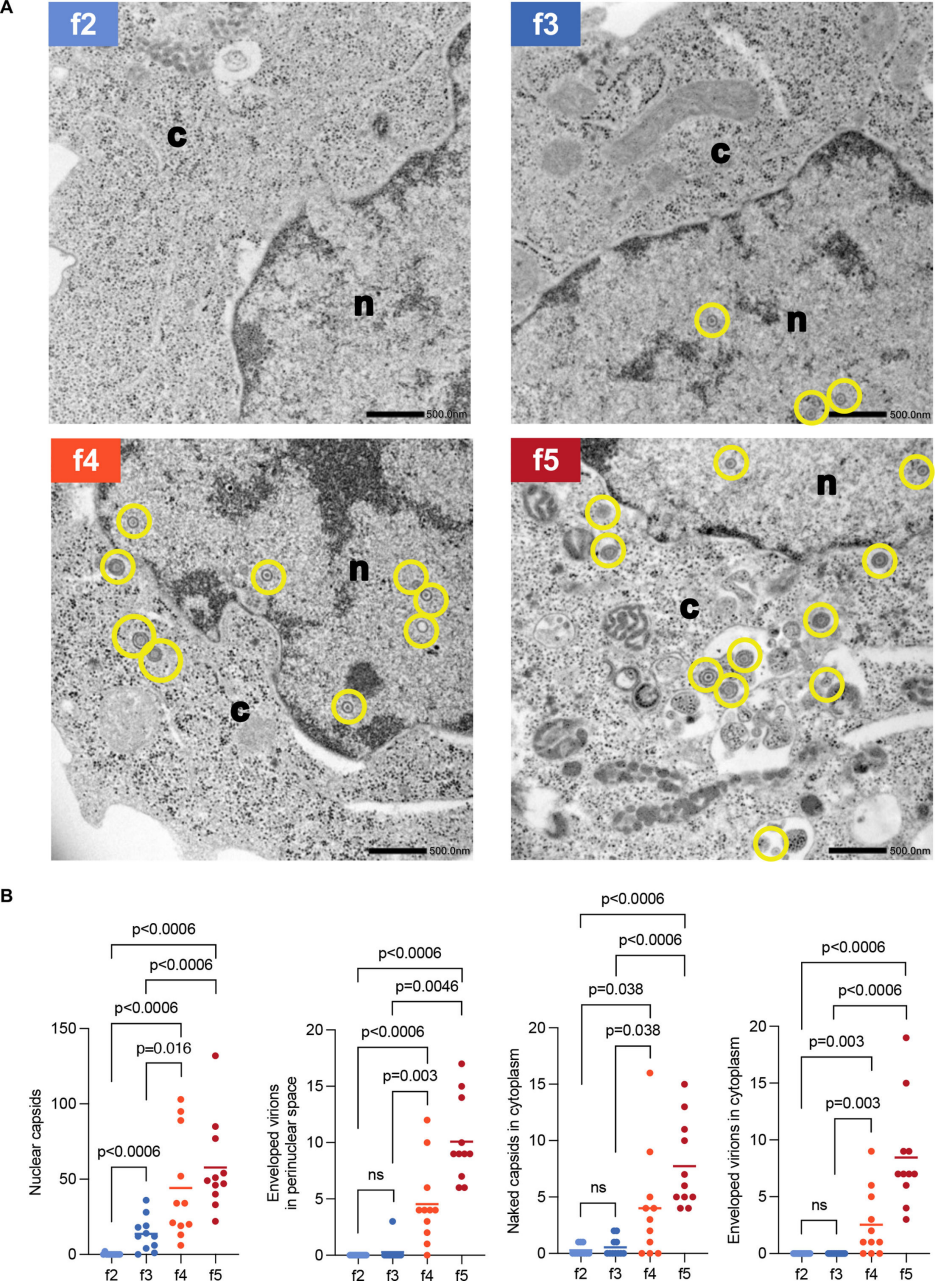
Electron microscopic analysis of cells in the f2–f5 subpopulations. (**A and B**) HeLa cells were infected with rICP47/vUs11 at an MOI of 5 and sorted into four subpopulations (f2–f5) by cell sorting 24 h after infection. Sorted cells were fixed, embedded, sectioned, stained, and examined by electron microscopy. (**A**) A transmission electron microscopy image of cells in the f2–f5 subpopulations. n, nucleus; c, cytoplasm. Scale bars = 500 nm. (**B**) The numbers of nuclear virions, enveloped virions in the perinuclear space, naked capsids in the cytoplasm, and enveloped virions in the cytoplasm of 11 cells in the f2–f5 subpopulations were quantitated. The horizontal bars indicate the means. Virions are marked in yellow. Statistical analysis was performed by the Mann-Whitney *U*-test, and *P*-values were adjusted by Holm correction. n.s., not significant. Raw data for these analyses are provided in Table S1C.

At the entire population level, virus titers were decreased 6 h after infection due to an eclipse phase of infection, and viral replication entered the productive phase between 6 and 8 h post-infection (Fig. 4A and C). These results indicated that infectious progeny virus production was detectable 8 h after infection in these experiments. The f4 subpopulation emerged at this time point (Fig. 3 and 4D, F, and G; Table S1B). Although 4.1% of total cells were in the f4 subpopulation 8 h after infection (Fig. 4D; Table S1B), 55% of progeny infectious virus yields were produced by this subpopulation (Fig. 4E; Table S1B). The f5 and f6 subpopulations emerged 10 and 12 h after infection, respectively, when the growth rate had slowed (Fig. 3 and 4D, F, and G; Table S1B). At 10 h after infection, 18% of total cells were in the f4 and f5 subpopulations (Fig. 4D; Table S1B), and these subpopulations produced 87% of progeny infectious virus yields (Fig. 4E; Table S1B). At 12 and 24 h post-infection, 33% and 53% of total cells were in the f4–f6 subpopulations, respectively (Fig. 4D; Table S1B), and these subpopulations produced 95% of progeny infectious virus yields (Fig. 4E; Table S1B). Similarly, most progeny infectious virus yields were produced by the f4–f6 subpopulations in HeLa cells using MOIs of 1 and 2.5 at 24 h post-infection and in other cells (Vero, U2Os and HaCaT cells, and human fetal foreskin fibroblasts [HFFF-2]) at MOIs of 1 or 2.5 at 12 h post-infection, although the proportion of each subpopulation varied by different MOI and cell type (Fig. S3B through U). Notably, the viral growth curve from 8 h post-infection based on the virtual titers using only the data of the f4–f6 subpopulations was almost identical to that based on the virtual titers using the data of the f1–f6 populations or actual titers of the entire population (Fig. 4H and I). Similarly, virtual titers using only the data of the f4–f6 subpopulations in HeLa cells at MOIs of 1 and 2.5 and in Vero, U2OS, and HaCaT cells, and HFFF-2 were also similar to virtual titers using the data of f1–f6 populations or actual titers in the entire population (Fig. S3V through AB). These results indicated that subpopulations f4–f6 have a predominant role in yielding progeny infectious viruses, whereas subpopulations f1–f3 barely produced infectious virions. To obtain evidence to further support this conclusion, we analyzed virion morphogenesis by quantitating the number of virus particles at different morphogenetic stages in HSV-1-infected HeLa cells at an MOI of 5 for 8 (Fig. S4) or 24 h (Fig. 5) in each subpopulation f2–f4 or f5, respectively, by electron microscopy. All virion types were barely detectable in infected cells from subpopulation f2 (Fig. 5; Fig. S4). In subpopulation f3, although nuclear virions were obvious, cytoplasmic virions, and especially enveloped virions in the cytoplasm that were considered infectious, were barely detectable (Fig. 5; Fig. S4). In contrast, enveloped virions were detected in the cytoplasm of 100% of infected cells in the f4 subpopulation at 8 h post-infection (Fig. S4), and these virions were detected in the cytoplasm of 70% and 100% of infected cells in the f4 and f5 subpopulations, respectively, at 24 h post-infection (Fig. 5). The proliferative profiles of enveloped virions in the cytoplasm (Fig. 5; Fig. S4) were similar to those of infectious virus titers (Fig. S5A).

We noted that, even at 4 and 6 h post-infection, the virus titers in f1–f3 subpopulations were detectable at a maximum of 2.8 × 10^2^ PFU/10^4^ cells (Fig. 4G; Table S1B). Taken together with the series of observations above (Fig. 4D, E, H, and I and 5; Fig. S3, S4, and S5A) indicating that infected cells in subpopulations f1–f3 barely produced infectious virions, it was not likely that infectious virus titers in subpopulations f1–f3 detected ranging from 2.6 × 10^1^ to 9.3 × 10^2^ PFU/10^4^ cells (Fig. 4G; Table S1B) represented progeny virus yields of infected cells in f1–f3 subpopulations. Cells in the f1–f3 subpopulations were likely to be aborted infected cells, and their frequencies 24 h after infection were similar to those reported previously in HeLa cells (13, 25).

**Fig 6:**
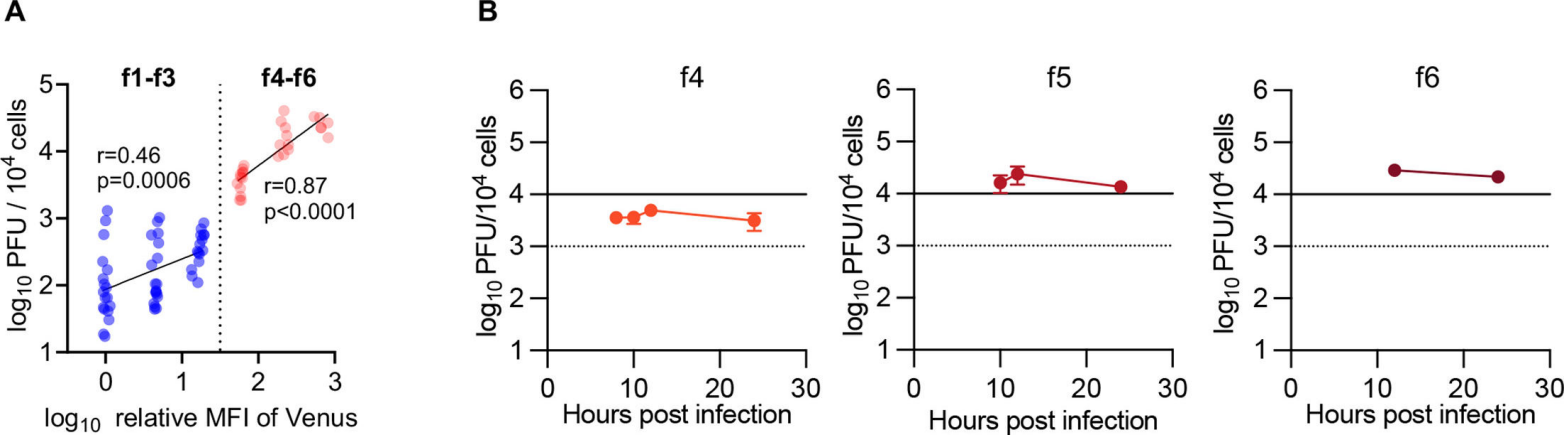
The f4–f6 subpopulations have a predominant role in yielding progeny infectious viruses. (**A**) Scatter plot of log_10_(relative mean fluorescent intensity [MFI] of Venus) vs log_10_(PFU/10^4^ cells) of subpopulations. (**B**) Progeny virus titers of the f4–f6 subpopulations at the indicated times after infection. Each value represents an individual value (**A**) or the mean ± SE (**B**) from three independent experiments. All data are obtained from the experiment shown in Fig. 4.

Collectively, these results, together with the observation that Venus-Us11 fluorescence intensities reflected the expression levels of global HSV-1 L proteins, indicated that certain levels of L protein expression were required for progeny virus production and that a threshold of progeny virus production existed between Venus-Us11 protein expression levels in subpopulations f3 and f4. Notably, Venus-Us11 fluorescence intensities in subpopulations above the threshold (f4–f6) correlated highly with progeny virus titers (*r* = 0.87; Fig. 6A). In contrast, a much lower correlation of coefficient (*r* = 0.46) was observed in subpopulations below the threshold (Fig. 6A). Furthermore, virus titers in subpopulations f4–f6 remained unchanged during HSV-1 infection (Fig. 6B; Fig. S5B), indicating that L protein expression levels in subpopulations above the threshold were tightly correlated with virus titers independent of the time point after infection. Thus, infectious progeny virus production in the entire population over time depended on the proportion of these subpopulations in the entire population.

### Virion morphogenesis with a defect in nucleocapsid maturation occurs in the subpopulation below the threshold for progeny virus production

**Fig 7:**
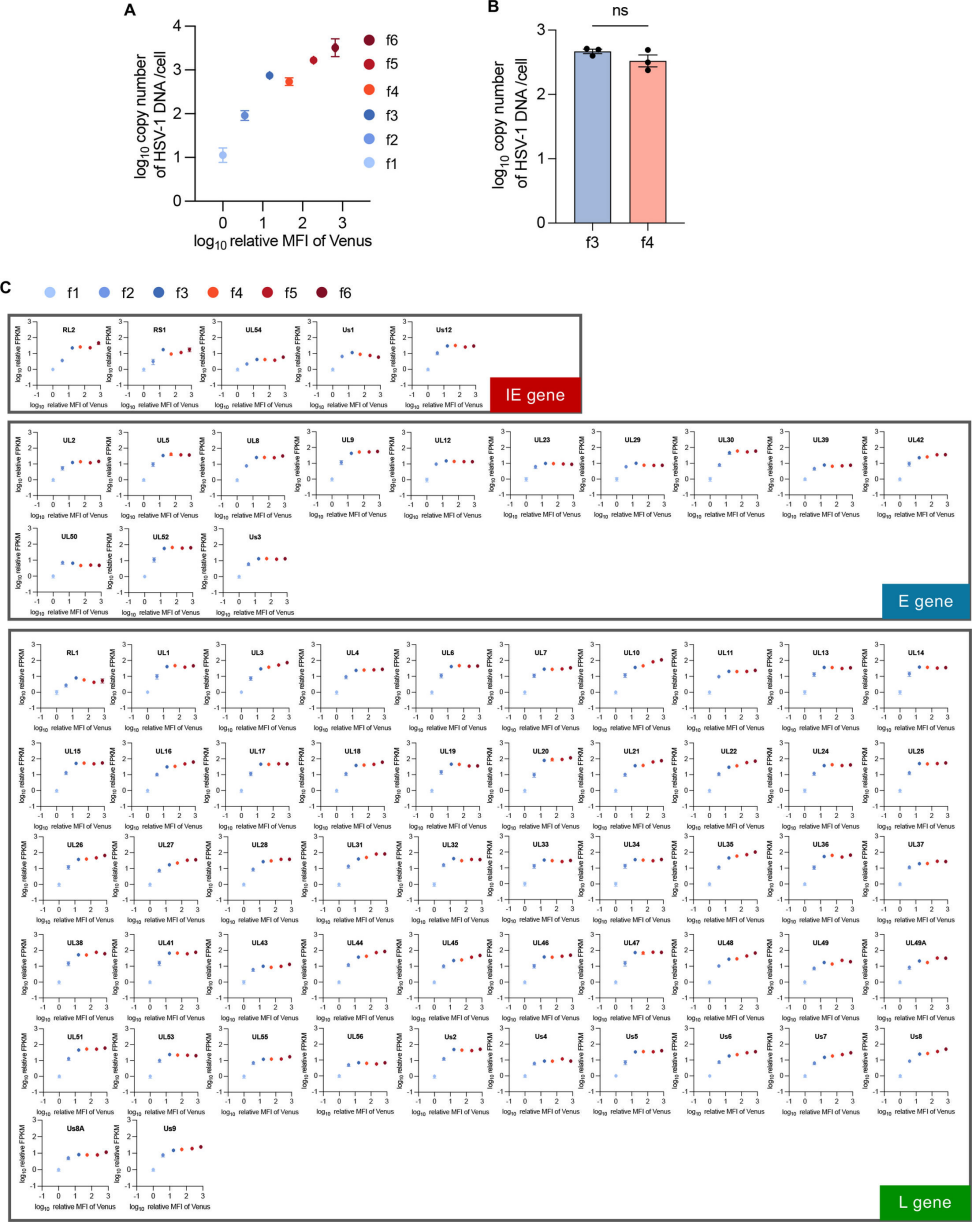
Amount of HSV-1 DNA and mRNA in the f1–f6 subpopulations. (A–C) HeLa cells were infected for 24 h with rICP47/vUs11 at an MOI of 5 and sorted into six subpopulations (f1–f6) by cell sorting. (**A and B**) Copy number of HSV-1 DNA in each subpopulation was analyzed by quantitative PCR. Panel B is a bar graph highlighting data from subpopulations f3 and f4 extracted from panel A. (**C**) Relative amounts of mRNA of selected genes in each subpopulation were analyzed by RNA-seq. Scatter plot of log_10_(relative mean fluorescent intensity [MFI] of Venus) vs log_10_(copy number of HSV-1 DNA) (**A**), or scatter plots of log_10_(relative MFI of Venus) vs log_10_(relative fragments per kilobase of exon per million mapped fragment [FPKM]) of the indicated HSV-1 genes (**C**) are shown. Each value is the mean ± SE of the results of three biologically independent samples. Statistical analysis was performed by unpaired Student’s *t*-test. ns, not significant (**B**).

The threshold for progeny virus production detected between Venus-Us11 protein expression levels in subpopulations f3 and f4 suggested a rate-limiting viral step for progeny virus production. Conceivably, this defect(s) might be observed in subpopulations below the threshold (f1–f3) but not in subpopulations above the threshold (f4–f6). To clarify this rate-limiting viral step, we compared levels of HSV-1 DNA genome replication and global viral mRNA expression in subpopulations f1–f6 by quantitative PCR and RNA sequencing, respectively. The copy number of HSV-1 genome DNA increased 57.8-fold from the f1 to f3 subpopulations and further increased 5.2-fold from the f3 to f6 subpopulations (Fig. 7A). However, there was no significant difference in the HSV-1 genome copy number between subpopulations f3 and f4 (Fig. 7B). Similar proliferative profiles were observed for expressions of each of the 70 HSV-1 mRNAs tested (Fig. 7C). Thus, we did not detect any rate-limiting effects of HSV-1 genome replication or viral mRNA expression associated with the threshold for progeny virus production.

**Fig 8:**
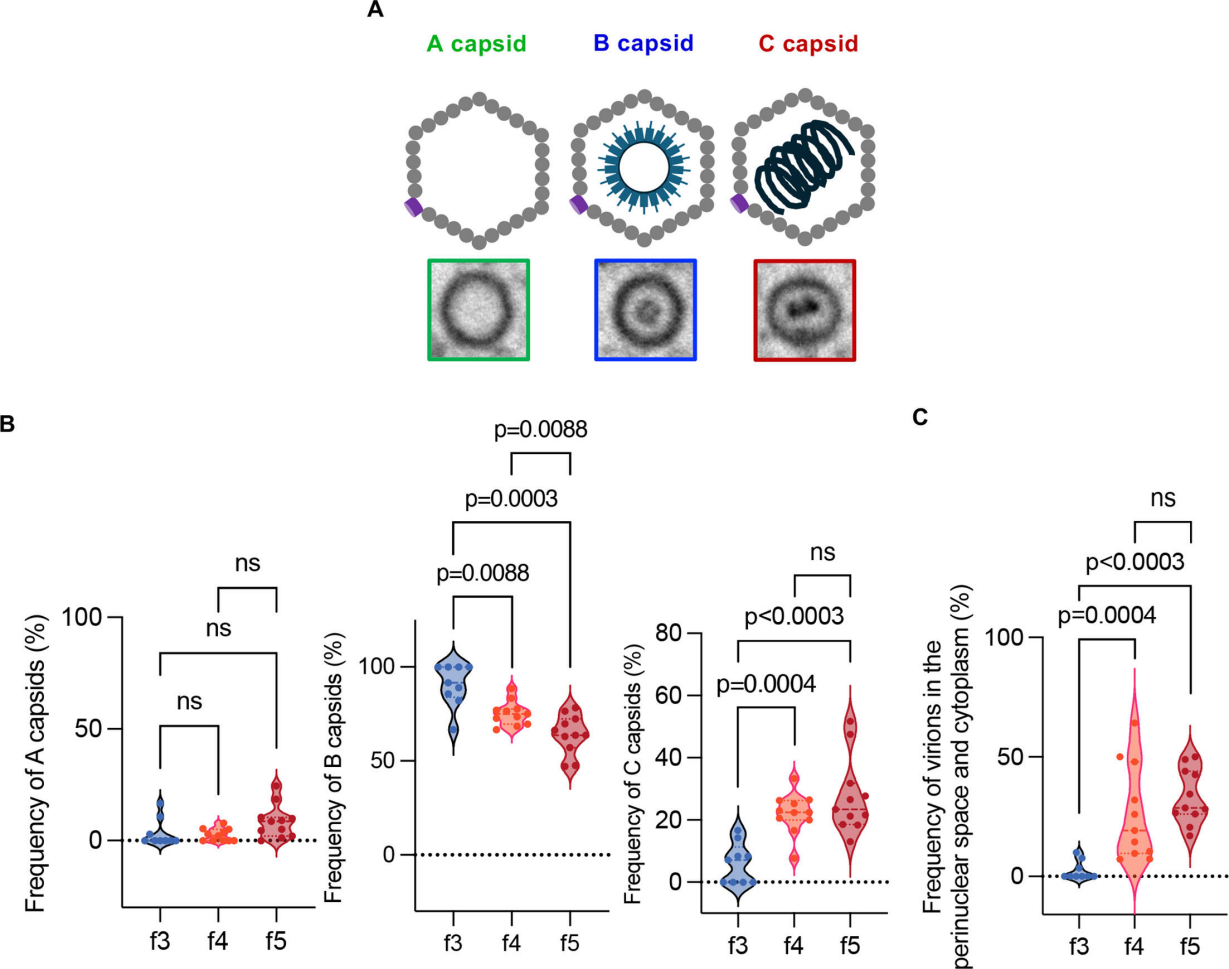
Frequencies of A, B, and C capsids in the nuclei of cells in the f2–f5 subpopulations. (**A**) Schematics of A, B, and C capsids. In the nucleus of HSV-1-infected cells, three distinct types of capsids are observed by electron microscopy: A capsids, which are empty; B capsids, which contain an internal protein scaffold; and C capsids, which encapsulate the viral genome. (**B and C**) A, B, and C capsids in the nuclei of 11 cells from the f2 to f5 subpopulations, analyzed in Fig. 5, were quantitated. (**B**) Proportions of A, B, and C capsids to nuclear capsids (**B**), or virions in the perinuclear space and cytoplasm to total virions (**C**) of cells with more than two nuclear capsids (f3, *n* = 9; f4, *n* = 11; f5, *n* = 11). Data are presented as the median ± interquartile range. Statistical analysis was performed by the Mann-Whitney *U*-test, and *P*-values were adjusted by Holm correction. ns, not significant. Raw data for these analyses are provided in Table S1C.

Next, we visualized virion morphogenesis by electron microscopy focusing on the A, B, and C capsids in the nucleus (Fig. 8; Fig. S6; Table S1C). The A and B capsids are incomplete structures resulting from problems in viral DNA genome retention in the capsids and packaging into the capsids, respectively (22–24). C capsids are mature capsids (nucleocapsids) containing viral DNA genomes reported to be selectively exported to the cytoplasm (26–29). In electron microscopy micrographs, these three types of capsids can be distinguished based on their appearance. A capsids are empty, B capsids contain an internal protein scaffold, and C capsids house the viral genome (Fig. 8A) (22, 23). As shown in Fig. 8B, Fig. S6, and Table S1C, B capsids accumulated aberrantly in subpopulation f3, and most (90.1%) nuclear capsids in this subpopulation were B capsids. The frequency of B capsids in subpopulation f3 was significantly higher than in subpopulations f4 and f5 (Fig. 8B). In contrast, the frequency of C capsids in subpopulation f3 was significantly lower than in subpopulations f4 and f5 (Fig. 8B). In agreement with this and previous reports that C capsids are selectively exported to the cytoplasm (26–29), the frequency of virions in the perinuclear space and cytoplasm in subpopulation f3 was significantly lower than in subpopulations f4 and f5 (Fig. 8C). The frequency of A capsids in subpopulation f3 was comparable to those in subpopulations f4 and f5 (Fig. 8B). The frequencies of each type of nuclear capsid in subpopulations f4 and f5 were similar to those reported previously in wild-type HSV-1-infected HeLa cells (30). Furthermore, the aberrant accumulation of B capsids, along with the lack of nuclear C capsids and virions in the perinuclear space and cytoplasm in subpopulation f3, was also observed at 8 h post-infection (Fig. S7; Table S1D). These features of subpopulation f3 were similar to the phenotypes of HSV-mutants including those lacking the portal protein UL6, a terminase subunit (UL15, UL33, or UL28), a minor capsid protein (UL17), or packaging accessory factor (UL32) (31–37). These mutants exhibited defects in viral DNA genome cleavage/packaging, characterized by the accumulation of B capsids and non-cleaved concatemeric viral genome DNAs, as well as a reduction in C capsids and viral genome DNAs packaged into capsids (31–41). Collectively, these results suggested that nucleocapsid maturation lacked in subpopulations below the threshold and that this viral step may be one of the rate-limiting steps for progeny virus production.

## DISCUSSION

Single-cell analyses have identified new subpopulations of virus-infected cells with similar viral gene expression profiles (15–17). However, the use of single-cell analyses to investigate both viral gene expression and progeny virus production is limited. Thus, single-cell analyses for both viral RNA synthesis and progeny virus production have been reported for some RNA viruses (10, 12, 14); a recent study reported that influenza virus transcription and progeny virus production were poorly correlated at the single-cell level (14), and other studies did not focus on the relationship between viral transcription and virus progeny production at the single-cell level (10, 12). Furthermore, protein expression and virus progeny production at the single-cell or subpopulation level have not been investigated to date. This study established a system to separate HSV-1-infected cell populations into multiple subpopulations based on global HSV-1 L protein expression levels and to titrate progeny virus yields in these fractions. This established system enabled us to clarify, for the first time, the direct and quantitative relationship between viral protein expression and progeny virus yields, and the clarified relationship indicated a threshold for HSV-1 L protein expression levels for progeny virus production. Such a threshold has not been reported by classical time course studies of most viruses at the entire population level by analyzing the relationship between viral gene expression and progeny virus yield. In contrast, once the levels of HSV-1 L protein expression exceeded the threshold, they were highly correlated with infectious progeny virus yields as reported by our (Fig. 4B) and other previous studies at the entire population level (4, 6).

**Fig 9:**
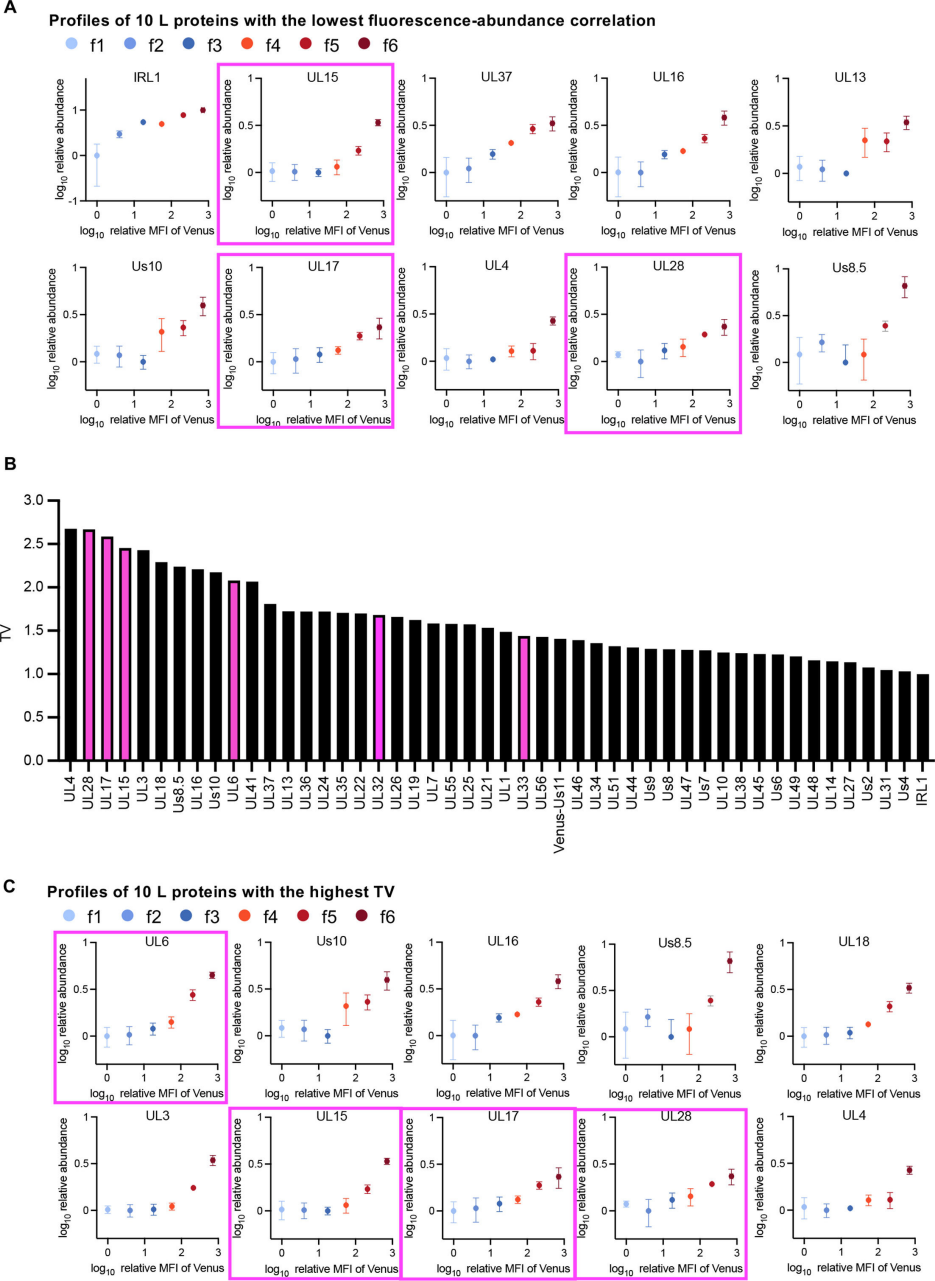
Unique protein expression profiles of a subset of HSV-1 L proteins. (**A**) Scatter plots of log_10_(relative mean fluorescent intensity [MFI] of Venus) vs log_10_(relative abundance) of the 10 HSV-1 L proteins (from Fig. S2A) with the lowest correlation coefficients between the fluorescence intensity of Venus and their abundance (Fig. S2A). Plots of proteins essential for DNA genome cleavage/packaging are marked with magenta squares. (**B**) TV of 48 L proteins (from Fig. S8), where TV is defined as the x-axis value at which the y-axis value increased by log_10_(2) (indicating a twofold increase in linear scale) from the starting point (*x* = 0) for each curve. Bars of proteins essential for DNA genome cleavage/packaging are highlighted in magenta. (**C**) Scatter plots of log_10_(relative MFI of Venus) vs log_10_(relative abundance; from Fig. S2A) of the top 10 HSV-1 L proteins with the highest TV (from panel B). Plots of proteins essential for DNA genome cleavage/packaging are marked with magenta squares. Each value is the mean ± SE (**A and C**) of the results of three independent experiments.

The threshold clarified in this study suggested that the nucleocapsid maturation step may be one of the rate-limiting steps for progeny virus production. Notably, the features of virion morphogenesis in subpopulations below the threshold were similar to the phenotypes of HSV mutants with defective viral DNA genome cleavage/packaging, including those lacking the portal protein UL6, a terminase subunit (UL15, UL33, or UL28), a minor capsid protein (UL17), or packaging accessory factor (UL32) (31–41). These results suggest that the cleavage/packaging of HSV-1 DNA genome was not sufficiently initiated or was specifically lacking in subpopulations below the threshold. Interestingly, the abundance profiles for several HSV-1 L proteins with the lowest correlation to the fluorescence intensity of Venus-Us11, including Us8.5, UL28, UL4, UL17, Us10, UL13, and UL15 (Fig. 9A), differed from those of other HSV-1 L proteins whose abundance continuously increased in subpopulations f1–f6 as shown in S-Fig. 2A. The abundance of these L proteins in subpopulations f1–f3 was very low and remained relatively constant but was increased in subpopulations f4–f6. To quantitatively evaluate the delayed increase in L protein abundance across subpopulations, we applied a four-parameter logistic regression to curve-fit these profiles at 24 h post-infection (Fig. S8). In this study, we introduced a novel indicator, the threshold value (TV), which was defined for each curve as the x-axis value at which the y-axis value increases by log₁₀(2) (equivalent to a twofold increase on a linear scale) from the baseline (*x* = 0; Fig. S8; Fig. 9B). Figure 9C showed the top 10 proteins with the highest TV included the subunits of HSV-1 terminase, UL15 and UL28, the portal protein UL6, and the minor capsid protein UL17, all of which were reported to be required for viral DNA genome cleavage/packaging (31, 35, 36, 40–43) (Fig. 9B and C). These results indicated that the expressions of a fraction of L proteins, including the subunits of HSV-1 terminase, the viral portal protein, and the minor capsid protein, were lacking in subpopulations f1–f3, unlike that of other L proteins. This suggested that the lack of these viral proteins might lead to a defect in the cleavage/packaging of HSV-1 DNA genome. It is also of interest that the expression profiles of this subset of L mRNAs were similar to those of other L mRNAs at the subpopulation level, suggesting that this absence might be mediated by post-transcriptional and post-translational mechanisms such as the regulation of mRNA stability, translational efficiency, and protein stability as reported previously (44). Notably, the newly established system is simple and widely applicable for studies of the direct relationship between viral gene expression and progeny virus yield of many other DNA and RNA viruses, which might provide specific and conserved insights into mechanisms of progeny virus production in these viruses. It will be particularly interesting to determine whether the rate-limiting step identified in the progeny virus production of HSV-1 is conserved in adenoviruses, which seem to utilize similar DNA genome packaging systems (45).

It has long been recognized that many fewer virions are present in the cytoplasm of HSV-1-infected cells compared with the nucleus (46–48). Our interpretation that the nucleocapsid maturation step may be one of the rate-limiting steps for progeny virus production is in agreement with these observations as well as earlier reports demonstrating C capsids are selectively exported to the cytoplasm (26–29). The rate-limiting step identified in this study also suggests a sophisticated strategy of efficient progeny virus production by HSV-1 without accumulating progeny nucleocapsids in the cytoplasm, which enables evasion from cytosolic sensors related to innate immune responses. In the cytoplasm, HSV-1 nucleocapsids are degraded by proteasomes (49), and viral DNAs are likely to be sensed by cytosolic sensors including cGAS, AIM2, DAI, and RNA polymerase III, which promote innate immune responses (50–55). Here, we showed that, in subpopulation f3, which was likely slightly below the threshold, HSV-1 genome DNA replication had already slowed, and B capsid formation was easily observed, although C capsid formation was barely detectable. In contrast, in subpopulation f4, which was likely slightly over the threshold, C capsids and all types of virions in the perinuclear space and the cytoplasm were evident. These observations suggested that downregulating the HSV-1 DNA cleavage/packaging might promote the accumulation of nucleocapsid components including capsids and viral genome DNAs in the nucleus as well as other viral proteins required for virion maturation at the nuclear membranes (NMs) and in the cytoplasm at a level sufficient for efficient progeny virus production. Once the viral genome is packaged into a capsid, the following steps for virion maturation might immediately proceed without accumulating any excess nucleocapsids in the cytoplasm, decreasing the chance for HSV-1 to be sensed by innate immune responses in the cytoplasm. IFI16 and hnRNPA2B1 sense HSV-1 genome DNA and promote innate immune responses in the nucleus (56–58). However, replicated HSV-1 genome DNAs and nucleocapsids appear to accumulate in the nucleus of HSV-1-infected cells (19, 59, 60), suggesting this virus has evolved more effective evasion mechanisms against innate immune responses in the nucleus such that ICP0 counteracts IFI16 (56) and probably, against capsid degradation than those in the cytoplasm.

We noted that overall viral mRNA levels increased from the f1 subpopulation and almost reached a plateau between subpopulations f2 and f3 (Fig. 7B). In contrast, viral L protein levels continued to increase from the f1 to f6 subpopulations (Fig. S2A). Thus, there was a lack of correlation between viral L mRNA levels and viral L protein levels, especially in fractions f4–f6. In agreement with these observations, previous systematic studies quantifying mRNA and protein levels at the genomic scale reported the importance of multiple processes beyond the mRNA concentration that contributed to establishing the level of a protein (44). These processes include translation rates, translation rate modulation, modulation of a protein’s stability, protein synthesis delay, and protein transport (44). Interestingly, it was reported that HSV-1 VP22 promoted global viral protein expression (61, 62), potentially by reducing the dependence of protein synthesis upon cellular ribosomal proteins to support translation during infection (63) and by promoting the translocation of viral L mRNAs to the cytoplasm (64). HSV-1 might evolve VP22 to promote global viral translation efficiency in specific circumstances such as in subfractions f4–f6, in which amounts of viral mRNAs are saturated.

There might be other possibilities involved in the rate-limiting step(s) for progeny virus production. One possibility is that HSV-1 proteins, which are significantly more abundant in subpopulations above the threshold compared to those below the threshold, act as triggers for progeny virus production. Among the HSV-1 proteins with significantly increased expression in the f3–f4 subpopulation, the expressions of Venus-Us11 and UL25 viral proteins were increased more than twofold (Table S1E). Us11 and UL25 were reported to suppress host immune responses (65, 66) and promote the packaging of HSV-1 DNA genome into a capsid (67), respectively. These activities of the HSV-1 proteins might serve as triggers for progeny virus production. Another possibility is that host cellular factors with significantly differential expression between the two subpopulations (Table S1F) trigger progeny virus production, possibly by regulating virion maturation or host immune responses. Furthermore, it has been well established that IE and E proteins regulate L gene expression (18). Therefore, there is a possibility that HSV-1 IE and/or E proteins might be indirectly involved in progeny virus production by regulating the expression of L proteins necessary for virus production. Further studies are needed to address these possibilities and are currently underway.

## MATERIALS AND METHODS

### Cells and viruses

HeLa, U2OS, HFFF-2, HaCaT, and Vero cells were described previously (20, 68). Wild-type HSV-1(F) was described previously (69).

### Generation of a recombinant virus

Recombinant virus YK410 (rICP47/vUs11) in which ICP47 and Us11 were tagged with TagRFP (70) and VenusA206K (71), respectively, was generated by two rounds of two-step Red-mediated mutagenesis using *Escherichia coli* GS1783 containing pYEbac102Cre (69, 72), a full-length infectious HSV-1(F) clone, pBS-Venus-KanS (20), pFlag-TagRFP-KanS, and the primers listed in Table S1A. The viruses used in this study were propagated and titrated in Vero cells.

### Infection procedure

HeLa, U2OS, HFFF-2, HaCaT, and Vero cells were seeded 1 day prior to infection. The following day, the cells were adsorbed with the recombinant virus YK410 (rICP47/vUs11) or wild-type HSV-1(F) at an MOI specified for each experiment in medium 199 containing 1% fetal calf serum (199V) for 1 h at 37°C. After adsorption, the medium was replaced with a fresh 199V medium. The cells were then incubated at 37°C in a CO_2_ incubator until the designated time points for analysis.

### Plasmids

pFlag-TagRFP was constructed by amplifying the TagRFP open reading frame (ORF) to introduce a *Kpn*I site without changing the amino acid sequence by PCR from pTagRFP-N1 (73) and cloning it into pFlag-CMV2 (Sigma). pFlag-TagRFP-KanS, used in the two-step Red-mediated mutagenesis procedure, was constructed by amplifying the domain of pEP-KanS (74) carrying the I-*Sce*I site and the kanamycin resistance gene by PCR from pEP-KanS using the primers 5′-GCGGTACCGTGAACAACCACCACTTCAAAGGATGACGACGATAAGTAGGG-3′ and 5′-GCGGTACCCTCCATGTACAGCTTCATGTCAACCAATTAACCAATTCTGATTAG-3′ and cloning it into the *Kpn*I site of pFlag-TagRFP. pGEX-ICP47 was constructed by amplifying the ICP47 ORF by PCR from the HSV-1(F) genome and cloning it into pGEX-4T-1.

### Antibodies

Commercial antibodies used in this study included a mouse monoclonal antibody to α-tubulin (DM1A; Sigma) and rabbit monoclonal antibodies togreen fluorescent protein (GFP) (598; Medical & Biological Laboratories [MBL]) and TagRFP (AB233; Evrogen). Rabbit polyclonal antibodies to US11 were described previously (47). To generate a rabbit polyclonal antibody to ICP47, a rabbit was immunized, according to the standard protocol at MBL, with GST-ICP47 expressed in *E. coli* and purified as described previously (20). Serum from the immunized rabbit was used as the anti-ICP47 polyclonal antibody.

### Immunoblotting

Immunoblotting was performed as described previously (75).

### Flow cytometry

HeLa cells infected with HSV-1(F) or YK410 (rICP47/vUs11) were washed with phosphate-buffered saline (PBS) and detached with 0.25% trypsin/EDTA solution (Wako). Then, the cells were suspended in PBS containing 2% fetal calf serum (FCS) (washing buffer), filtered through a 35 µm pore-cell strainer (#352235, Corning), and subjected to fluorescence-activated cell sorting (FACS) analysis with BD FACS Melody (Becton Dickinson). The data were analyzed with BD FACSChorus (Becton Dickinson) software or FlowJo 10.8.1 software (Becton Dickinson).

To compare the kinetics of Us11 expression in HSV-1(F)- and rICP47/vUs11-infected cells, HeLa cells were infected at an MOI of 5. At 4, 6, 8, 10, 12, and 24  h post-infection, cells were detached with trypsin, washed once with washing buffer, fixed with 4% paraformaldehyde in PBS, and permeabilized with 0.1% Triton X-100 in PBS. The cells were then incubated with rabbit polyclonal anti-Us11 antibody in washing buffer for 2 h at room temperature, washed, and further incubated with Alexa Fluor 647-conjugated anti-rabbit IgG (Invitrogen) for 1 h at room temperature. After another wash, the cells were analyzed using a CytoFLEX S flow cytometer (Beckman Coulter), and data were processed with FlowJo 10.8.1 software (Becton Dickinson). For each sample, the mean fluorescent intensity (MFI) of uninfected cells was subtracted from the MFI to obtain the Us11 signal. The relative Us11 signal was then calculated by expressing each sample’s Us11 signal as a fraction of the total Us11 signal across all samples in the experiment. Finally, the data were normalized so that the mean relative Us11 signal of HSV-1(F)-infected cells at 4  h post-infection (across the three independent experiments) was set to 1 before plotting.

### Gating strategy based on Venus-Us11 fluorescence intensity for cell sorting

The gating strategy for sorting cells infected with rICP47/vUs11 into six subpopulations was defined using HeLa cells infected with rICP47/vUs11 at an MOI of 5, as follows. The subpopulation of Venus-negative cells was defined as the f1 subpopulation. The f6 subpopulation containing infected cells with the highest Venus fluorescence intensity was defined as the minimal range of infected cells in which Venus fluorescence was consistently detectable at 12 and 24 h post-infection. The remaining fluorescence intensity range was evenly divided into four intervals, and subpopulations f2–f5 were defined to include infected cells within each of these evenly divided intervals. The gating strategy as defined above was applied constantly to all other experiments involving the binning of infected cells.

### Determination of viral titer in the subpopulations separated by cell sorting

HeLa cells were infected with YK410 (rICP47/vUs11) at an MOI of 5. At the indicated times after infection, cells were detached and suspended as described above. Then, 1.5 × 10^4^ to 3 × 10^4^ cells in the f1–f6 subpopulations and the entire cell population (FSC singlet) were sorted into medium 199 containing 1% FCS by FACS Melody (Becton Dickinson). The sorted cells were freeze-thawed once and sonicated, and virus titers were determined by plaque assay using Vero cells. The average virus titer per single cell was obtained by dividing the obtained virus titer by the number of sorted cells. Data are presented as the PFU per 10^4^ cells.

### Calculation of the proportion of virus titers in each subpopulation and the virtual titer

PFU_fi_, which represents the viral titer of each subpopulation fi (*i* = 1–6), was obtained using the following equation:


$$PFU_{fi}=PFU_{fi/cell}\times 10^{4}\times PR_{fi}/100$$

where PFU_fi/cell_ is the average viral titer per single cell belonging to the fi subpopulation (from Fig. 4F and G), and PR_fi_ is the proportion (%) of cells in the fi fraction in the entire population (from Fig. 4D). The virtual titer (VT), which represents the sum of PFU_fi_, was obtained as follows:


$$VT=PFU_{f1}+PFU_{f2}+PFU_{f3}+PFU_{f4}+PFU_{f5}+PFU_{f6}$$

% of PFU_fi_, which represents the proportion of viral titers in the fi subpopulation accounted for in the entire population (Fig. 4E), was obtained using the following equation:


$$\%\;of\;PFU_{fi}=PFU_{fi}/VT\times 100$$

### LC-MS/MS analysis of the subpopulations separated by cell sorting

HeLa cells were infected with rICP47/vUs11 at an MOI of 5 for 24 h. Then, cells were detached and suspended as described above. Next, 10^5^ cells in the f1 to f6 subpopulation were sorted into PBS by BD FACS Melody (Becton Dickinson), pelleted by centrifugation, and lysed with lysis buffer (0.1 M of Tris-HCl [pH 8.0], 1% SDS). To remove SDS from samples, we used the methanol-chloroform protein precipitation method. Briefly, we added 4 vol of methanol, 1 vol of chloroform, and 3 vol of water to the eluted sample and mixed thoroughly. The samples were centrifuged at 15,000 rpm for 10 min, the water phase was carefully removed, and then 4 vol of methanol was added to the samples. The samples were centrifuged at 15,000 rpm for 10 min, and the supernatant was removed. The pellet was washed once with 100% ice-cold acetone. The precipitated protein was re-dissolved in guanidine hydrochloride, reduced with TCEP, alkylated with iodoacetamide, and digested with lysyl endopeptidase and trypsin. The resulting digested peptides were analyzed using an Evosep One LC system (EVOSEP) connected to a Q-Exactive HF-X mass spectrometer (Thermo) with a Dream spray tip (AMR) and a 15 cm × 150 µm column packed with 1.9 µm C18-beads (Evosep). The mobile phases were composed of 0.1% FA as solution A and 0.1% FA/99.9% ACN as solution B. The analysis was performed in the data-dependent acquisition mode, where the top 25 recorded mass spectrometry spectra between 380 and 1,500 *m*/*z* were selected. Survey scans were acquired at a resolution of 60,000 at *m*/*z* 200, and the tandem mass spectrometry (MS/MS) resolution was set to 15,000 at *m*/*z* 200. All MS/MS spectra were searched against the protein sequences of the HSV protein database and human Swiss-Prot database using Proteome Discoverer 2.2 (Thermo) with the SEQUEST search engine, and the result of HSV proteins was extracted. The false discovery rate was set to 1% on peptide spectrum match.

### Data processing of LC-MS/MS data

Normalization was performed on quantitative values using the method built into the Discoverer 2.2 software. We used the “Peptide Amount Mode” within the software to normalize between samples. In this mode, the peptide group abundances for each sample were summed, and the maximum sum across all samples was determined. The normalization factor for each sample was calculated as the ratio of the maximum sum across all samples to its sum, and the abundances were adjusted accordingly. The resulting values were treated as normalized abundances. Subsequently, we performed a scaling step on the normalized abundances. This scaling was applied separately to each of the three biological replicates. In this method, we adjusted the abundance values in each sample so that the average abundance across all samples was 100. Specifically, we calculated the average of all normalized abundances across all samples in a biological replicate and then scaled the abundance values in each sample proportionally so that this average equaled 100. The values obtained from this step were considered scaled abundances. When a peptide was undetected in all four technical replicates from a sample of each subpopulation, the protein was considered undetectable in that sample. If a peptide was undetected in only one (or some) of the technical replicates, the missing value was replaced with 0. The mean scaled abundance from the four technical replicates was used as the value for one experiment. The relative abundance of each HSV-1 L protein was determined by normalizing its mean abundance from three biologically independent experiments to the subpopulation with the lowest mean abundance, which was set as the reference value of 1.

### RNA sequencing in the subpopulations separated by cell sorting

HeLa cells were infected with YK410 (rICP47/vUs11) at an MOI of 5 for 24 h. Then, cells were detached and suspended as described above. Next, 5 × 10^5^ cells in the f1–f6 subpopulations were sorted into PBS by BD FACS Melody (Becton Dickinson). After centrifugation, total RNA from fractionated cells was isolated with a High Pure RNA Isolation kit (Roche). (i) Each cDNA was generated using a Clontech SMART-Seq HT Kit (Takara Clontech, Mountain View, CA, USA), and each library was prepared using a Nextera XT DNA Library Prep Kit (Illumina, San Diego, USA). Sequencing was performed on the DNBSEQ-G400 platform in the 100 + 100 base paired-end mode. (ii) Generated reads were a mixture of human reads and HSV-1 reads. For human data, generated reads were mapped to the human (hg19) reference genome using TopHat v2.1.1 combined with Bowtie2 ver. 2.2.8 and SAMtools ver. 0.1.18. For HSV-1, generated reads were mapped to the HSV-1 (GenBank: GU734771.1) reference genome using HISAT2 v2.1.0. (iii) The quantification to obtain the read counts of each gene from HSV-1 was performed using featureCounts of subread-2.0.0 with option -M --fraction. With the length data of each gene (another output of the featureCounts), fragments per kilobase of exon per million mapped fragments (FPKMs) were calculated according to the definition. The FPKM of HSV-1 genes was multiplied by (total number of mapped reads of the HSV-1 genome)/[(total number of mapped reads of the human genome) + (total number of mapped reads of the HSV-1 genome)], normalized by the amount relative to the value of the f1 fraction.

### Quantification of HSV-1 DNA in the subpopulations separated by cell sorting

HeLa cells were infected with YK410 (rICP47/vUs11) at an MOI of 5 for 24 h. Then, cells were detached and suspended as described above. Next, 5 × 10^5^ cells in the f1–f6 subpopulations were sorted into PBS by BD FACS Melody (Becton Dickinson). After centrifugation, total DNA from fractionated cells was isolated with NucleoSpin Tissue XS (TAKARA) according to the manufacturer’s instructions. The amount of HSV-1 DNA was quantified using the Universal Probe Library (Roche) with TaqMan Master (Roche) and the LightCycler 96 System (Roche) according to the manufacturer’s instructions. Primers and probes targeting the region encoding ICP27 in the HSV-1 genome were designed using the probe finder software. The sequences for the primers and probe used to detect ICP27 (HSV-1 DNA) were as follows: forward primer 5′-TCCGACAGCGATCTGGAC-3′, reverse primer 5′-TCCGACGAGGAACACTCC-3′, and Universal ProbeLibrary Probe #56. The HSV-1 genome copy numbers were quantified using quantitative PCR with these primers and probes. To determine the copy numbers, a standard curve was generated using the plasmid pFlag-CMV2-UL54 (54), which encodes ICP27.

### Electron microscopic analysis of the subpopulations separated by cell sorting

HeLa cells were infected with YK410 (rICP47/vUs11) at an MOI of 5 for 8 h or 24 h. Then, cells were detached and suspended as described above. Next, 5 × 10^5^ to 1 × 10^6^ cells in the f2–f4 (8 h) or f2–f5 (24 h) subpopulations were sorted by BD FACS Melody (Becton Dickinson). After centrifugation, the pelleted cells were examined by ultrathin-section electron microscopy as described previously (76) except using JEM-1400 Flash microscope (JEOL). Capsids were manually classified as A, B, or C type based on their morphological characteristics.

### Statistical analysis

The unpaired Student’s *t*-test was used to compare the two groups. One-way analysis of variance (ANOVA) followed by the Tukey multiple comparisons test, or Mann-Whitney *U-*test followed by Bonferroni correction, was used for multiple comparisons. A *P*-value < 0.05 was considered statistically significant. For the statistical comparison of viral titers, data were converted to common logarithms (log_10_). Pearson correlation coefficients (*r*) and *P*-values were calculated on log_10_-transformed data. GraphPad Prism 10 (GraphPad Software) was used to perform statistical analyses.

### Classification of HSV-1 genes

HSV-1 genes were classified into IE, E, and L genes as described previously (19).

### Curve fitting

To describe the abundance profiles of HSV-1 L proteins, we mainly used the following mathematical model developed by dose-response modeling (77).


(1) $$f\left(x\right)=c+\frac{d-c}{1+\exp\left[b\left(x-a\right)\right]}$$

The variable f(x) is the abundance profiles of HSV-1 L proteins. The parameter a is the inflection point of the curve for this model, that is, the point where a change in acceleration in the curve occurs. The parameter b is a slope parameter, and the parameters c and d are the lower and upper horizontal asymptotes or limits, respectively.

Fitting was performed by the drc package in R, which estimates the parameters with nonlinear least squares under the assumption of normally distributed response values (78).

## Data Availability

All data needed to evaluate the conclusions in the paper are present in the paper and/or the supplemental material. The mass spectrometry data were deposited in the Japan ProteOme STandard Repository (jPOST) under the ID JPST002328 and JPST003549. RNA-seq data have been deposited to Gene Expression Omnibus (GEO) with accession number GSE240449.

## SUPPLEMENTAL MATERIAL

Supplemental figures mbio.00280-25-s0001.pdf Figures S1 to S8.

Supplemental table mbio.00280-25-s0002.xlsx Table S1.

ASM does not own the copyrights to Supplemental Material that may be linked to, or accessed through, an article. The authors have granted ASM a non-exclusive, world-wide license to publish the Supplemental Material files. Please contact the corresponding author directly for reuse.
