# Supplementary material for: Direct relationship between protein expression and progeny yield of herpes simplex virus 1
Source: mBio. 2025 May 5;16(6):e00280-25. doi: 10.1128/mbio.00280-25 (PMC12153267; doi:10.1128/mbio.00280-25)
Supplement: Supplemental figures — Figures S1 to S8. [file mbio.00280-25-s0001.pdf]

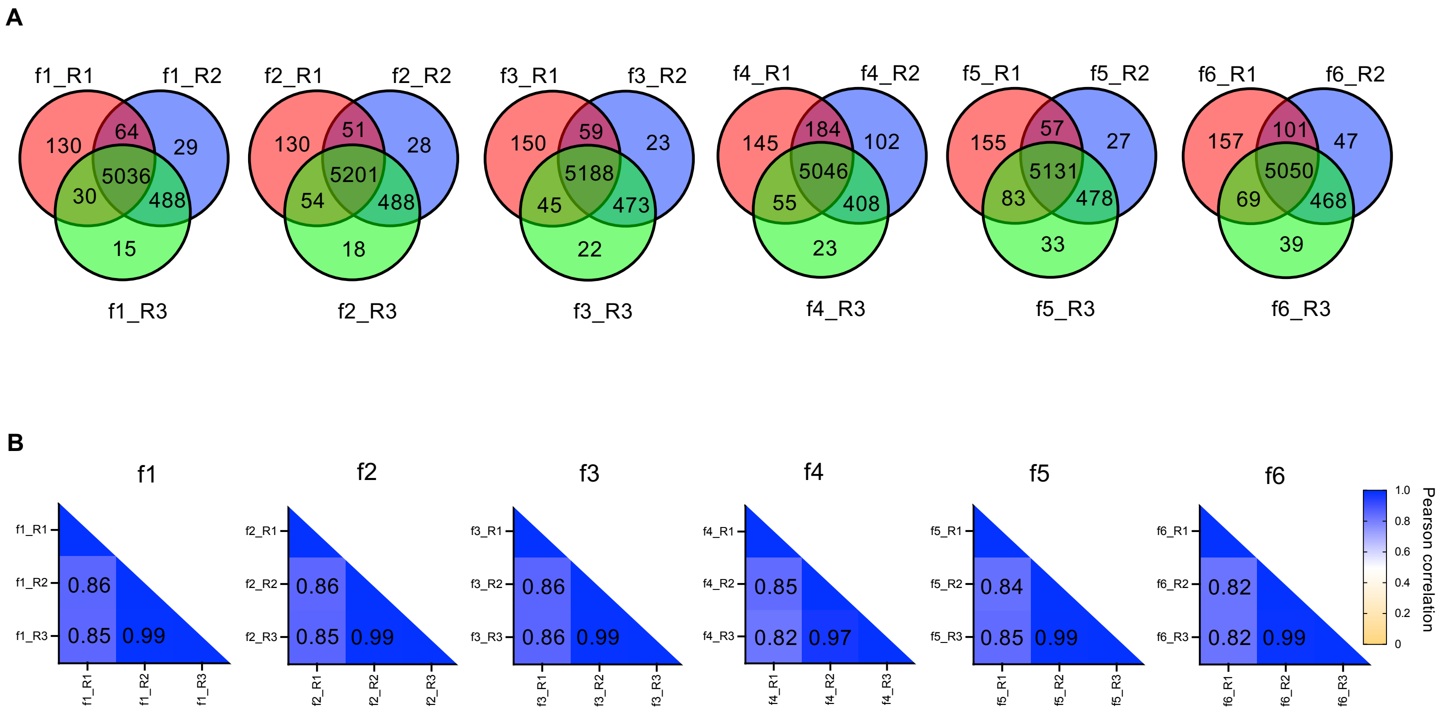

Supplementary Fig. 1. Reproducibility of LC-MS/MS analysis across biological replicates. (A) Venn diagrams showing the overlap of virus and host proteins identified in three biological replicates (R1, R2, R3) for each subpopulation. R1, Replicate 1; R2, Replicate 2; and R3, Replicate 3. (B) Heatmaps showing the pairwise Pearson correlation coefficients between biological replicates of protein abundances for each subpopulation.

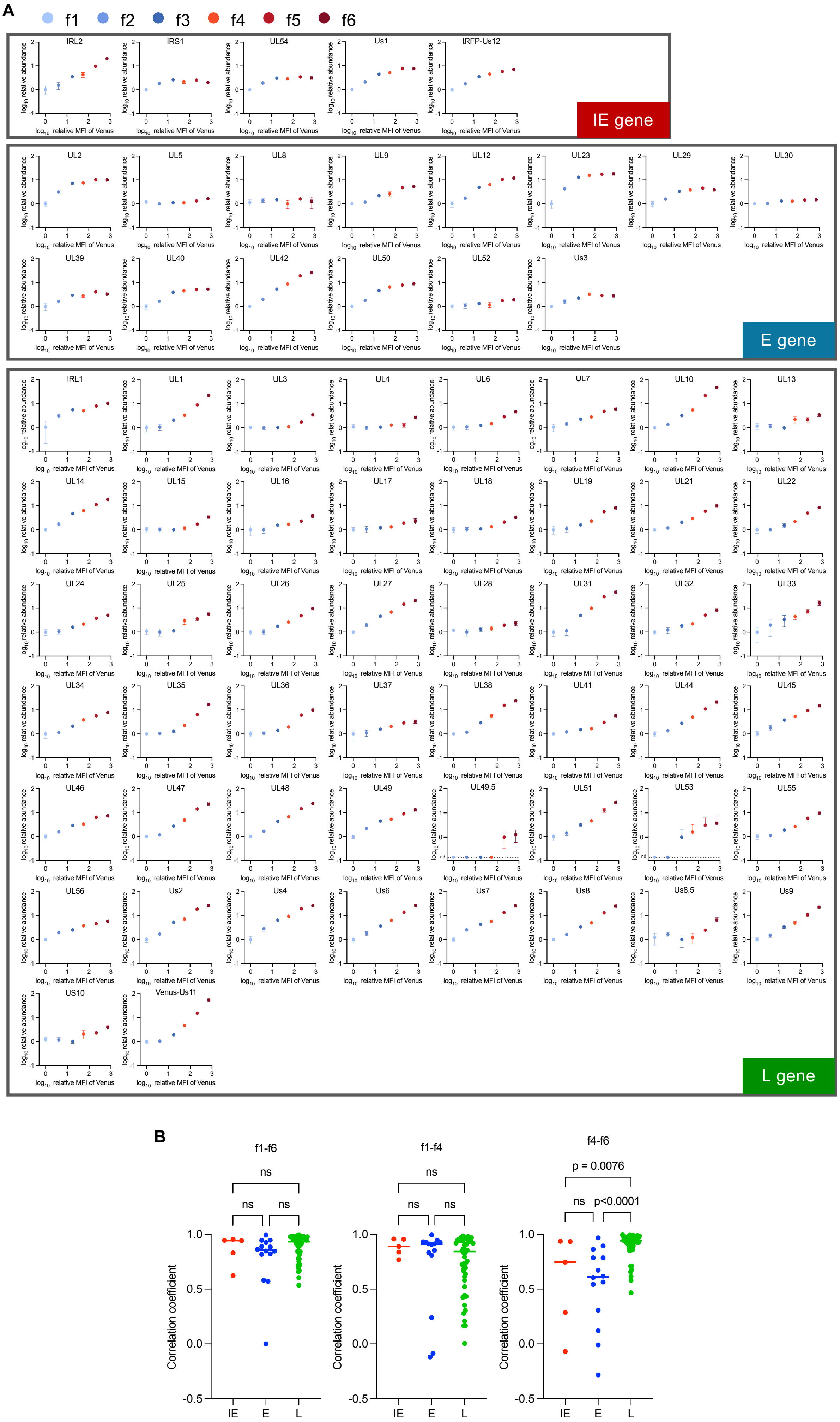

(Legend on next page)

Supplementary Fig. 2. Abundance of HSV-1 proteins in each subpopulation of rICP47/vUs11 infected cells. (A) Scatter plots of log_{10}(relative MFI of Venus) vs log_{10}(relative abundances) of the 5 indicated HSV-1 IE proteins, 14 indicated E proteins, and 50 indicated L proteins. Each value is the mean± standard error of the results of three biologically independent experiments. (B) Pearson correlation coefficient between log_{10} (MFI of Venus) and log_{10}(relative abundance) for 5 IE proteins, 14 E proteins, and 48 L proteins across subpopulations f1 to f6 and f4 to f6. Horizontal bars indicate the mean correlation coefficient for each protein group. Statistical analysis was performed by one-way ANOVA followed by Tukey's test. n.s., not significant.

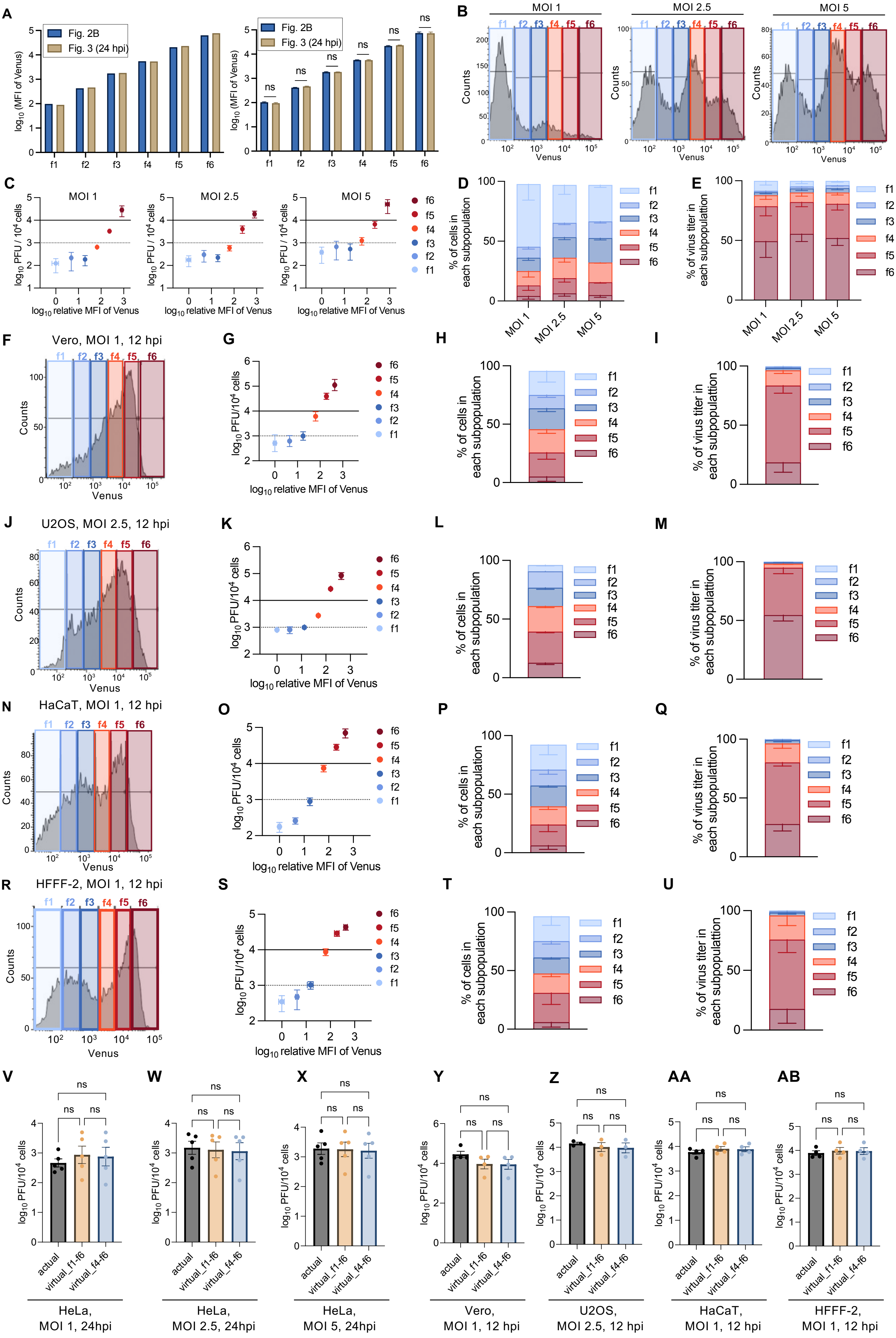

(Legend on next page)

Supplementary Fig. 3. Quantitative analysis of the relationship between the expression levels of HSV-1 L proteins and progeny virus yields across different MOIs and cell lines. (A) Bar graphs showing the raw mean fluorescence intensity (MFI) of Venus-Us11 in f1 to f6 subpopulations. The left panel presents data from the experiments shown in Fig. 2B and Fig. 3 (24 hpi) as a representative experiment, while right shows the mean ± standard error from three independent experiments. (B to E) HeLa cells infected with rICP47/vUs11 at the indicated MOI for 24 h, were sorted into f1 to f6 subpopulations (B), sonicated and virus titers were determined by plaque assay using Vero cells. (C) Scatter plot of $\log_{10}$(relative MFI of Venus) vs $\log_{10}$(PFU/10^4 cells). (D) Proportion of cells in the indicated subpopulations account for the entire cell population at the indicated MOIs. (E) Proportion of virus titers produced by the f1 to f6 subpopulations account for virus titers produced by the entire population at the indicated times after infection. Vero (F to I), U2OS (J to M), HaCaT (N to Q), or HFFF-2 (R to U) cells infected with rICP47/vUs11 at an MOI of 1 (F to I, and N to U) or 2.5 (J to M) for 12 h, were sorted into f1 to f6 subpopulations (F, J, N, and R), sonicated, and virus titers were determined by plaque assay using Vero cells. (G, K, O, and S) Scatter plots of $\log_{10}$(relative MFI of Venus) vs $\log_{10}$(PFU/10^4 cells) of each subpopulation. (H, L, P, and T) Proportion of cells in the f1 to f6 subpopulations account for the entire cell population. (I, M, Q, and U) Proportion of virus titers produced by each subpopulation account for the virus titers produced by the entire cell population. (V to AB) Progeny virus titers of the entire population (actual) and sum of the virus titers produced by f1 to f6 (virtual_f1-f6) or f4 to f6 (virtual_f4-f6) subpopulations under the indicated infection conditions. The data are from experiments (B) to (U). Each value is the mean ± standard error of the results of three (J to M and Z), four (F to I, N to Q, R to U, Y, AA, AB), or five (C to E and V to X) independent experiments. Solid and dashed lines indicate 1 PFU/cell and 0.1 PFU/cell, respectively (C, G, K, O, and S). Statistical analysis was performed by unpaired Student's t-test, with P-values adjusted by the Bonferroni correction (A), or one-way ANOVA followed by the Tukey test (V to AB). n.s., not significant

A

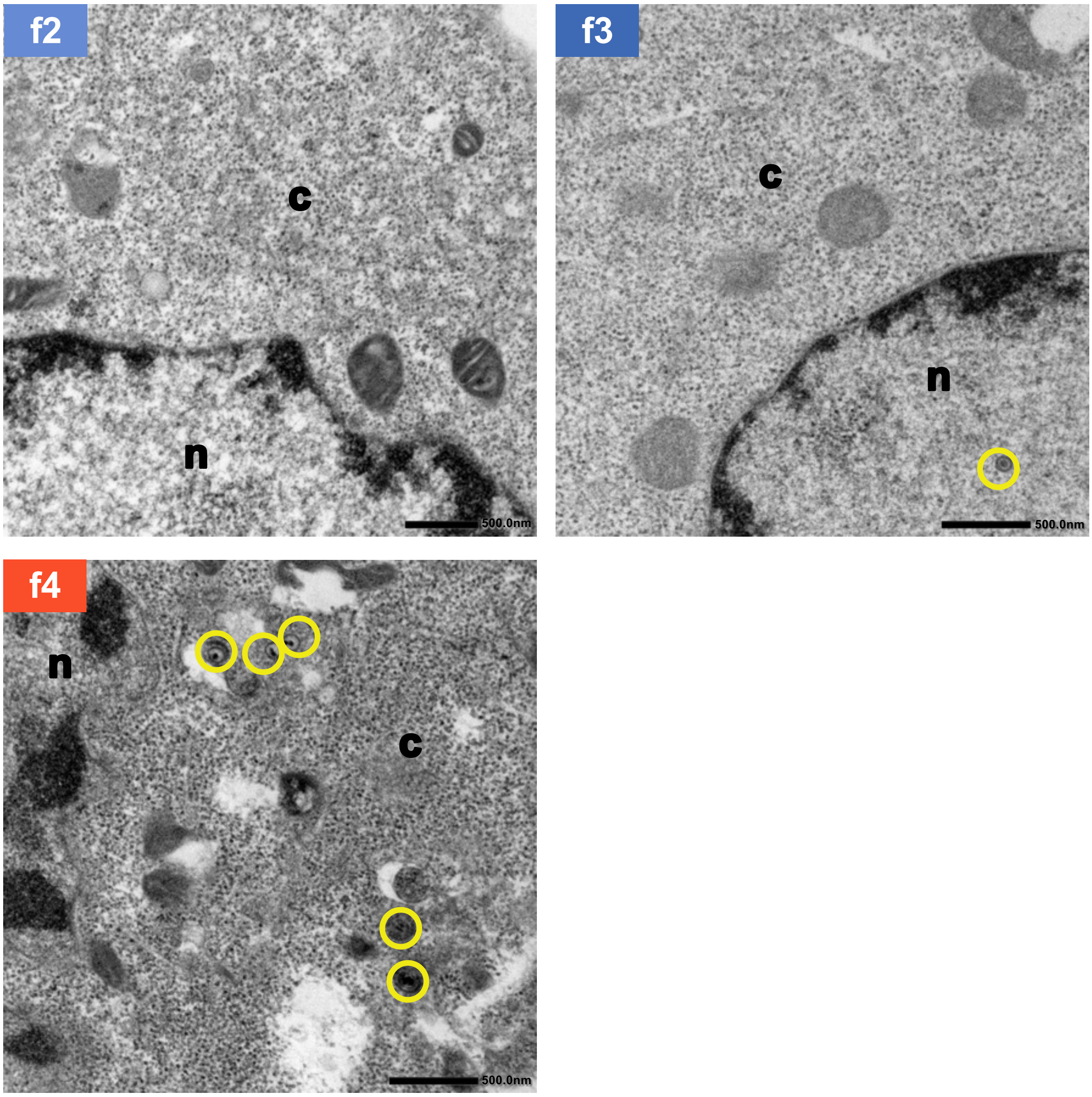

B

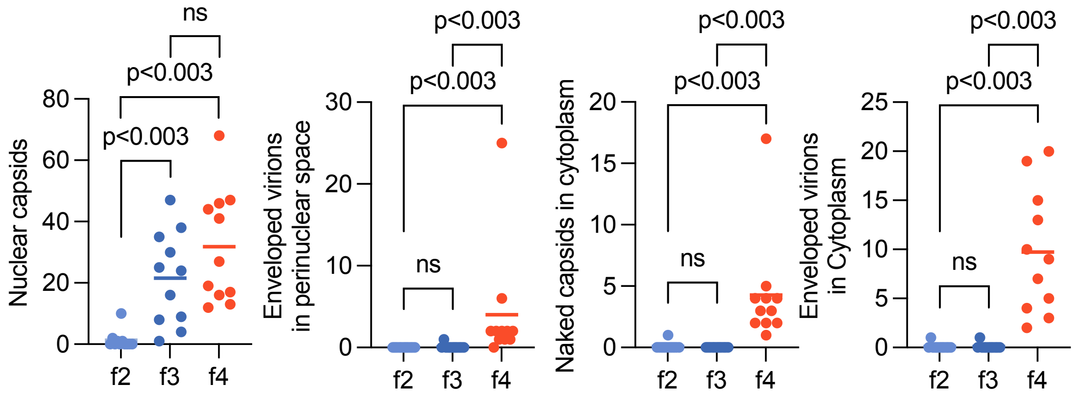

Supplementary Fig. 4. Electron microscopic analysis of cells in the f2 to f4 subpopulations at 8 h after infection. (A and B) HeLa cells were infected with rICP47/vUs11 at an MOI of 5 and sorted into three subpopulations (f2 to f4) by cell sorting 8 h after infection. Sorted cells were fixed, embedded, sectioned, stained, and examined by electron microscopy. (A) A transmission electron microscopy image of cells in the f2 to f4 subpopulations. n, nucleus; c, cytoplasm. Scale bar = 500 nm. (B) The numbers of nuclear virions, enveloped virions in the perinuclear space, naked capsids in the cytoplasm, and enveloped virions in the cytoplasm of 11 cells in the f2 to f4 subpopulations were quantitated. The horizontal bars indicate the means. Enveloped virions and naked capsids are marked in yellow. Raw data for these analyses are provided in S-Table 1D.

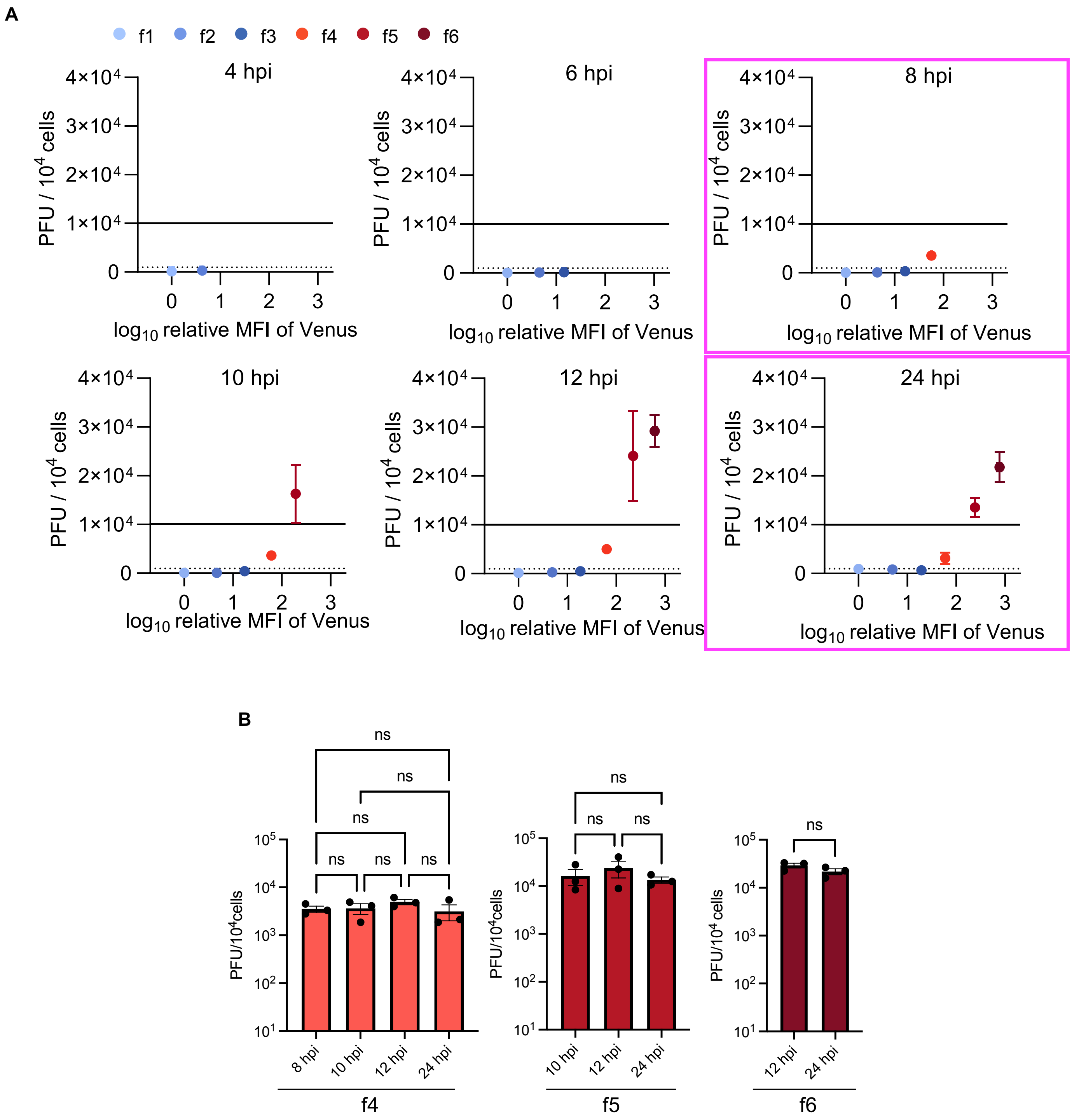

Supplementary Fig. 5. F4 to f6 subpopulations have a predominant role in yielding progeny infectious viruses. (A) Scatter plots of $\log_{10}$(relative MFI of Venus) vs PFU/10^4 cells of the indicated subpopulation separated by time after infection from Fig. 4 panel G. Scatter plots under the same conditions of electron microscopic analysis performed in Fig. 5 and S-Fig. 4 are shown in magenta. (B) Progeny virus titers of the f4 to f6 subpopulations at the indicated times after infection from Fig. 6 panel B. Statistical analysis was performed by one-way ANOVA followed by the Tukey test. n.s., not significant.

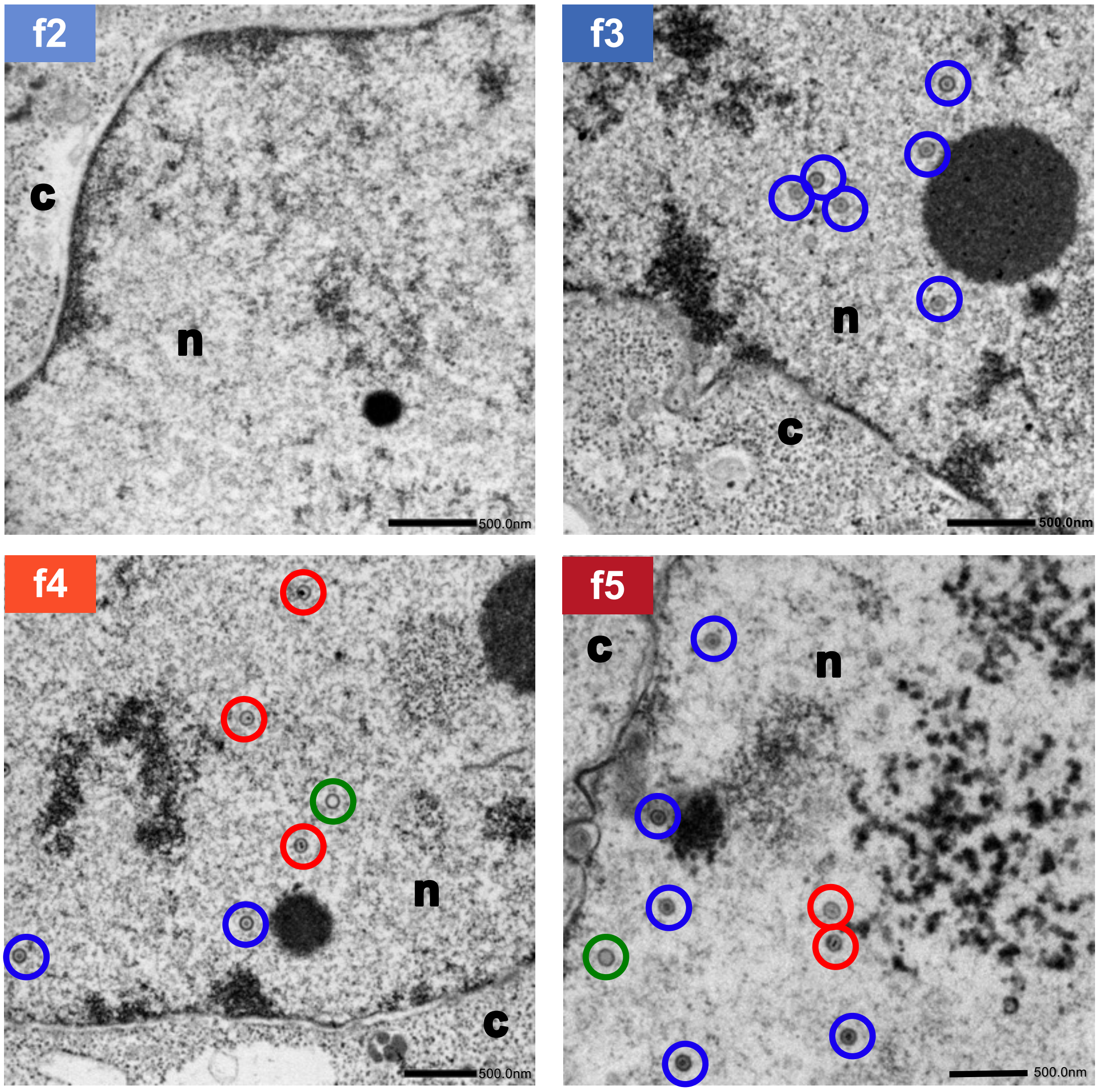

Supplementary Fig. 6. Frequency of A, B, and C capsids in the nuclei of cells in f2 to f5 subpopulations. A transmission electron microscopy image of cells in the f2 to f5 subpopulations in the experiment shown in Fig. 5. n, nucleus; c, cytoplasm. Type A capsids are marked in green, B capsids in blue, and C capsids in red. Scale bars = 500 nm.

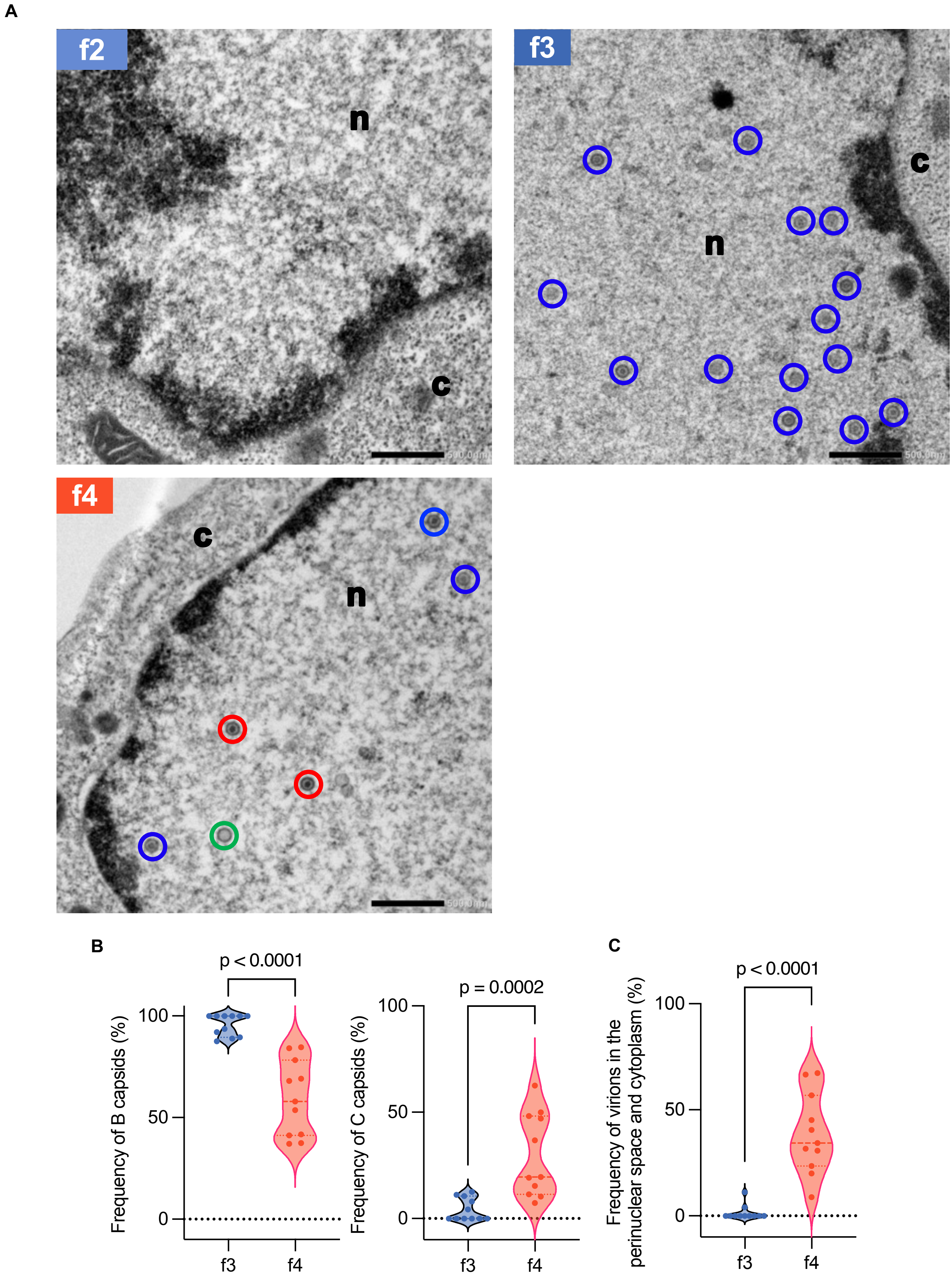

Supplementary Fig. 7. Frequencies of B and C capsids in the nuclei of cells in the f2 to f4 subpopulations at 8 h after infection. (A) A transmission electron microscopy image of cells in the f2 to f4 subpopulations in the experiment shown in S-Fig. 4. n, nucleus; c, cytoplasm. Type A capsids are marked in green, B capsids in blue, and C capsids in red. Scale bar = 500 nm. (B and C) A, B, and C capsids in the nucleus of 11 cells from the f2 to f4 subpopulations, analyzed in S-Fig. 4, were quantitated. Proportions of B and C capsids to nuclear capsids (B), or virions in the perinuclear space and cytoplasm to total virions (C) of cells with more than two nuclear capsids (f3, n = 11; f4, n = 11). Data are presented as the median ± interquartile range (IQR). Statistical analysis was performed by the Mann-Whitney *U*-test. Raw data for these analyses are provided in S-Table 1D.

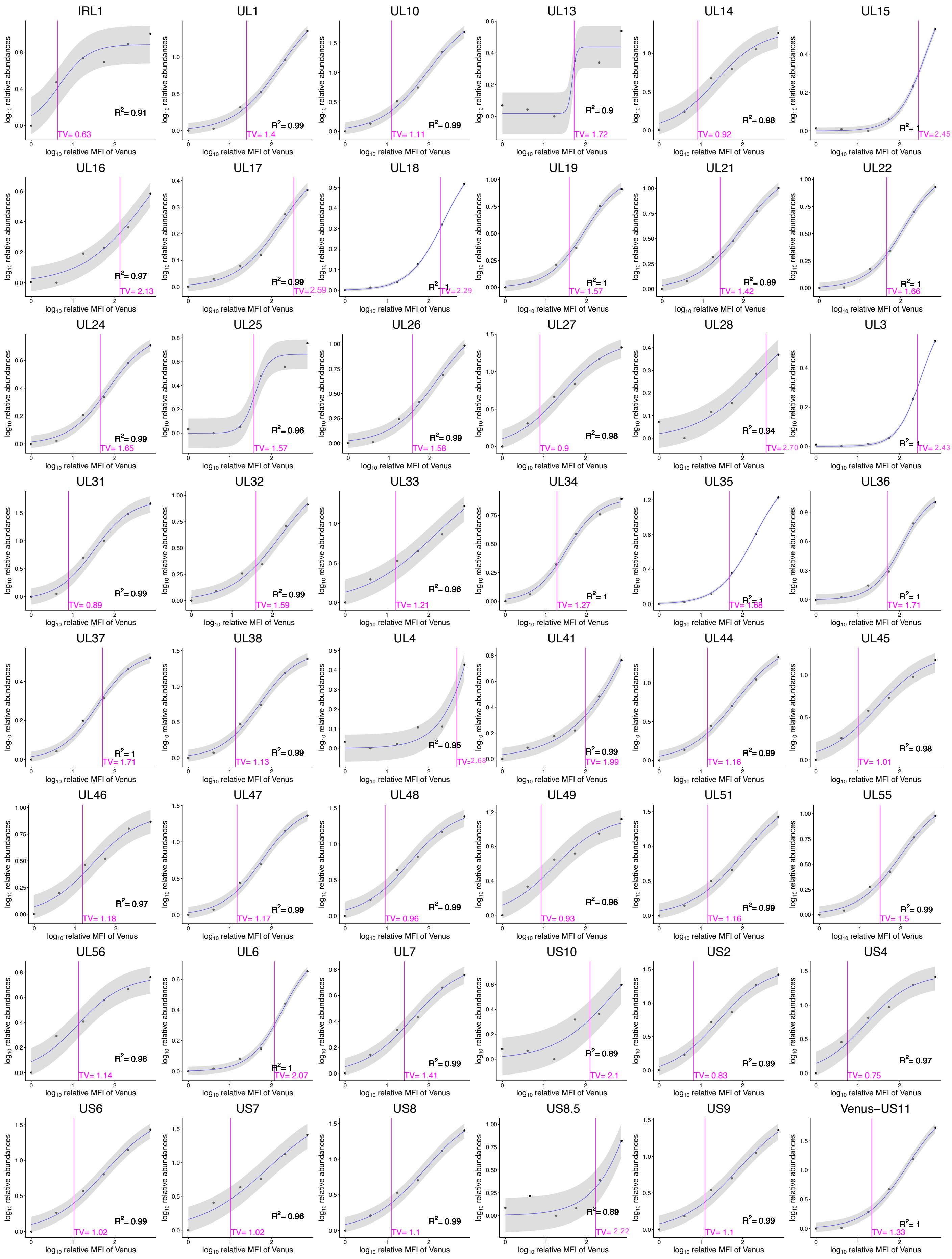

Supplementary Fig. 8. Curve fitting to abundance profiles of HSV-1 L proteins. Scatter plots of log_{10}(relative MFI of Venus) vs log_{10}(relative abundances) of the 48 indicated HSV-1 L proteins (from S-Fig. 2A) were fitted to a four-parameter logistic regression curve. TVs are the x-axis value at which the y-axis value increases by $log_{10}2$ (2-fold increase in linear scale) from the starting point (x = 0) for each curve. The grey regions correspond to 95% confidence intervals. The average value of three biologically independent experiments was used for curve fitting.
